# A Scoping Review of Machine-Learning Derived Radiomic Analysis of CT and PET Imaging to Investigate Atherosclerotic Cardiovascular Disease

**DOI:** 10.3390/tomography10090108

**Published:** 2024-09-03

**Authors:** Arshpreet Singh Badesha, Russell Frood, Marc A. Bailey, Patrick M. Coughlin, Andrew F. Scarsbrook

**Affiliations:** 1 Department of Radiology, St. James’s University Hospital, Leeds Teaching Hospitals NHS Trust, Leeds LS9 7TF, UK; 2 Faculty of Medicine and Health, University of Leeds, Leeds LS2 9TJ, UK; 3 The Leeds Vascular Institute, Leeds General Infirmary, Leeds Teaching Hospitals NHS Trust, Leeds LS1 3EX, UK

**Keywords:** radiomics, artificial intelligence, machine learning, cardiovascular disease, carotid, coronary, CT angiography, CT coronary angiography, PET, molecular imaging

## Abstract

Background: Cardiovascular disease affects the carotid arteries, coronary arteries, aorta and the peripheral arteries. Radiomics involves the extraction of quantitative data from imaging features that are imperceptible to the eye. Radiomics analysis in cardiovascular disease has largely focused on CT and MRI modalities. This scoping review aims to summarise the existing literature on radiomic analysis techniques in cardiovascular disease. Methods: MEDLINE and Embase databases were searched for eligible studies evaluating radiomic techniques in living human subjects derived from CT, MRI or PET imaging investigating atherosclerotic disease. Data on study population, imaging characteristics and radiomics methodology were extracted. Results: Twenty-nine studies consisting of 5753 patients (3752 males) were identified, and 78.7% of patients were from coronary artery studies. Twenty-seven studies employed CT imaging (19 CT carotid angiography and 6 CT coronary angiography (CTCA)), and two studies studied PET/CT. Manual segmentation was most frequently undertaken. Processing techniques included voxel discretisation, voxel resampling and filtration. Various shape, first-order, second-order and higher-order radiomic features were extracted. Logistic regression was most commonly used for machine learning. Conclusion: Most published evidence was feasibility/proof of concept work. There was significant heterogeneity in image acquisition, segmentation techniques, processing and analysis between studies. There is a need for the implementation of standardised imaging acquisition protocols, adherence to published reporting guidelines and economic evaluation.

## 1. Introduction

Cardiovascular disease (CVD) encompasses the pathology of the blood vessels, which most commonly affects the carotid arteries, coronary arteries, aorta and the peripheral arteries. The clinical sequalae of CVD include ischaemic heart disease, cerebrovascular disease, aortic aneurysms and peripheral arterial disease [1]. The prevalence of CVD has risen globally, from 271 million in 1990 to 523 million in 2019 [1]. The pathophysiology of CVD is multi-factorial and varies depending on the anatomical location of the vessel. However, in general, it is driven by maladaptive remodelling of the vessel wall as a consequence of hypertension, diabetes mellitus, hypercholesterolaemia, obesity and tobacco consumption [1,2].

The type of imaging employed in CVD varies depending on the disease process but includes duplex ultrasonography, fluoroscopic angiography, computed tomography angiography (CTA) or magnetic resonance angiography (MRA). These focus on the anatomy of vessels. In addition, molecular imaging can be employed using radiopharmaceuticals, which accumulate in vascular tissues to demonstrate active pathophysiological processes such as inflammation, angiogenesis or apoptosis [3]. Molecular imaging techniques include single-photon emission computed tomography (SPECT) and positron emission tomography (PET), which can be combined with CT or magnetic resonance imaging (MRI) for anatomical localisation and functional assessment. Molecular imaging is well established in oncology, with ^18^Fluorine-fluorodeoxyglucose (^18^F-FDG) PET imaging widely utilised to investigate primary and metastatic malignancy. Moreover, within CVD, SPECT imaging using ^99m^Technetium-sestamibi or ^99m^Tc-tetrofosmin radiotracers is clinically indicated for the investigation of myocardial perfusion in coronary artery disease [4]. Routine clinical use of radiopharmaceutical tracers within CVD is limited. Various radiolabelled tracers linked to an antibody, peptide or nanoparticle have been employed to investigate intracellular and extracellular targets in in vivo animal and human studies [3].

**Figure 1:**
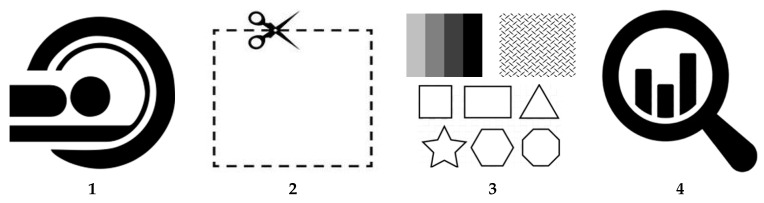
Methodological steps involved in radiomics. (**1**) Image acquisition. (**2**) Image segmentation. (**3**) Extraction of image features. (**4**) Analysis of quantitative data.

Radiomics involves the extraction of quantitative data from imaging features that are imperceptible to the eye [5]. These include, but are not limited to, texture, intensity and shape features. Due to the large quantities of data being handled, artificial intelligence techniques are necessary to extract and analyse the derived information. In particular, machine learning can be employed where programmed algorithms iteratively analyse the data to identify patterns in the information [6]. The utility of machine learning within radiomics can occur at the segmentation, image feature extraction and analysis stages, as outlined in Figure 1. 

The potential utility of radiomics has been reported primarily within the field of oncology. Radiomics enables the evaluation of heterogeneity in malignancy between patients, hence facilitating the assessment of tumour aggressiveness and prognosis [7]. In doing so, the treatment of different patients can be tailored accordingly, highlighting radiomics as a technique to support precision medicine. As imaging investigations play an integral role in the diagnosis and management of patients, it is anticipated that imaging databases can be formed to foster the creation of big data, which can facilitate large-scale radiomic analysis and enable more comprehensive evaluation of disease processes [7].

Radiomics has been evaluated in the CVD setting, largely focusing on CTA and MRA, with a relative paucity of publications exploring the utility of molecular imaging-derived radiomic analysis. Additionally, there is a lack of evidence summarising the literature in this field. Consequently, this scoping review aims to summarise the existing literature on radiomic analysis techniques using CT, MRI and PET imaging to investigate CVD. 

## 2. Methodology 

This scoping review was compiled in accordance with the Preferred Reporting Items for Systematic Reviews and Meta-Analyses for Scoping Reviews checklist [8]. A checklist has been included as Supplementary Table S1. This review acts as an antecedent to primary research into the utility of radiomic techniques to predict clinical outcomes in patients with peripheral arterial disease following angioplasty. Consequently, the included literature is aimed to facilitate an exploration into the methodology for such analysis through comparisons from work in carotid and coronary atherosclerotic disease using a range of imaging modalities.

### 2.1. Eligibility Criteria

Studies were eligible if the methodology involved the evaluation of radiomic techniques in living human subjects derived from CT, MRI or PET imaging being performed to investigate atherosclerotic disease [9]. The included studies focused on the detection or prediction of atherosclerotic CVD in the native carotid, coronary or lower limb peripheral arteries. Both prospective and retrospective studies were included and focused on original in vivo research. Only English language peer-reviewed articles were considered to facilitate data extraction and comprehension of the findings. Studies that only involved feasibility testing of radiomic techniques without assessing clinical outcomes such as adverse outcomes were excluded. Additionally, studies were ineligible if they involved cadaveric samples, non-native vessels such as stented vessels or other imaging modalities such as single-photon emission computed tomography (SPECT). Finally, studies were only included if the analysis involved machine learning techniques.

### 2.2. Sources and Search Strategy 

The Ovid platform was accessed to search MEDLINE and Embase databases [10]. The search strategy constituted three core areas with associated synonyms: (1) radiomics, (2) CT/MRI/PET and (3) atherosclerosis. Electronic search terms are summarised in the Supplementary Materials. Searches were conducted from the inception of the database until 11 April 2024 to maximise the number of records. Conference abstracts were excluded; however, targeted searches were performed to locate corresponding full-text studies. Additionally, the reference lists in systematic reviews and literature reviews were examined to identify additional eligible reports. Finally, searches were extended to Google Scholar to explore the grey literature for further salient research. Study titles and their corresponding abstracts were initially reviewed. Thereafter, the full text of pertinent studies was reviewed to assess their eligibility for inclusion. 

### 2.3. Data Extraction and Reporting 

Data on the study population (number of patients, age and gender); imaging characteristics (modality and imaging protocol) and radiomics methodology (software, segmentation technique, processing and performance evaluation) were extracted onto a data collection proforma by A. S. B. With respect to the segmentation methodology, automated segmentation was defined as the delineation of the region of interest using software algorithms without manual input, whereas semi-automated segmentation combined the automated process with manual input, such as adjusting the boundaries of the region of interest [11]. The METhodological RadiomICs Score [12] (METRICS) tool was used to develop the proforma and ensure that pertinent information on radiomics methodology was retrieved. 

Significant heterogeneity in study populations, imaging investigations and radiomics methodology precluded statistical analysis of the extracted data. Consequently, all findings were presented narratively in tables or the text. Where reported, *p*-values less than 0.05 were considered statistically significant, unless otherwise stated.

### 2.4. Quality Assessment

The Newcastle–Ottawa Scale [13] was used to assess the quality of the included studies. This tool was chosen as it enabled the evaluation of case–control studies and observational studies. The tool reviews the selection of study participants and outcomes, the comparability of cases and controls and the rigour of outcome assessment. Certain items were omitted from within the various parameters if they were irrelevant. Consequently, the relative scores differed between the studies.

## 3. Results

### 3.1. Literature Search 

**Figure 2:**
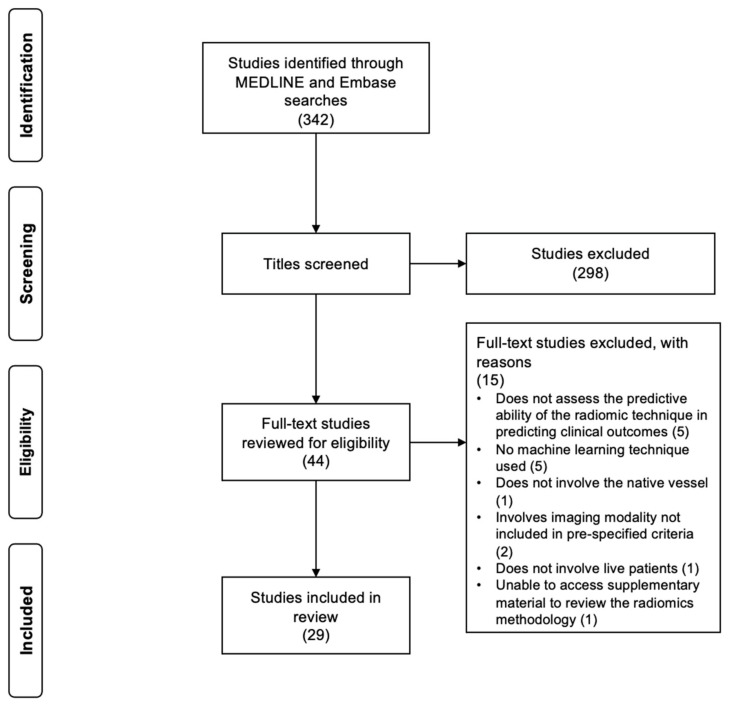
Preferred Reporting Items for Systematic Reviews and Meta-Analyses (PRISMA) flow diagram. Adapted from Moher et al. [43].

The literature search revealed 342 studies once duplicates were removed (Figure 2). Following the initial screening of titles and abstracts, 44 publications were selected for full review. Thereafter, 29 studies [14,15,16,17,18,19,20,21,22,23,24,25,26,27,28,29,30,31,32,33,34,35,36,37,38,39,40,41,42] fulfilled the eligibility criteria and were included in the scoping review (Table 1). Of these, seven studies [17,28,29,30,31,32,34] achieved maximum scores when assessed using the Newcastle–Ottawa Scale (Supplementary Table S2). Ten studies [14,15,26,36,37,38,39,40,41,42] focused on carotid imaging, whilst the remainder [16,17,18,19,20,21,22,23,24,25,27,28,29,30,31,32,33,34,35] explored radiomic analysis of the coronary arteries. No studies involving the peripheral arteries were found. The majority of research was conducted in a single centre; however, there were seven multi-centre studies [17,24,25,28,31,34,37]. Eleven studies [14,16,17,18,20,23,24,37,40,41,42] were published in 2023, eight studies [15,19,26,30,32,33,34,35] were published in 2022 and four studies [22,25,28,38] were published in 2024. The remaining studies were published prior to 2022 [21,27,29,31,36,39].

### 3.2. Study Characteristics

In total, 5753 patients were included, consisting of 3752 males (65.2%). There were more individuals enrolled into coronary studies (4529 patients, 78.7%) compared to carotid studies (1224 patients), although patients in the latter group were older, with a mean age ranging from 61 ± 8.0 years (±standard deviation) to 74.1 ± 8.4 years compared to 48.5 ± 11.6 years to 71.3 ± 7.8 years in the coronary studies. Hypertension (3482 patients, 60.5%), hyperlipidaemia (2278 patients, 39.6%) and tobacco smoking (2170 patients, 37.7%) were the three most observed comorbidities. 

Nine of the ten carotid studies included patients receiving clinically indicated investigations for carotid atherosclerotic disease, of which four specified the minimum degree of stenosis: 30% [14,42] or 70% [15,36], in accordance with the European Society of Vascular Surgery (ESVS) guidelines [47]. Seventeen [16,17,18,20,21,22,23,24,27,28,29,30,31,32,33,34,35] of the nineteen coronary studies included patients being clinically investigated for atherosclerotic disease. The exclusion criteria for the studies are outlined in Table 1. 

### 3.3. Image Acquisition 

In the carotid literature, CTA was reported as the technique of choice in eight studies [14,15,37,38,39,40,41,42] (Table 2 and Supplementary Table S3). Ebrahimian et al. [26] performed dual-energy CTA, whilst Kafouris et al. [36] undertook PET/CT imaging using ^18^F-FDG. No studies using MRI that fulfilled the inclusion criteria were identified.

A variety of iodine-based contrast agents were used, including iomeprol [15], iohexol [26,37], ioversol [38], iopamidol [39] and iopromide [42]. In the contrast studies, the tube voltage ranged from 80 kV [40] to 120 kV [39,41,42], whilst the tube current ranged from 100 mA [40] to 320 mA [26]. A variety of slice thicknesses were employed ranging from 0.5 mm [37,40] to 1 mm [26], whilst the slice interval ranged from 0.4 mm [39] to 0.625 mm [42]. 

All of the coronary studies evaluated CTA, with the exception of Kwiecinski et al., who reported outcomes in PET/CT imaging using ^18^fluorine-sodium fluoride (^18^F-NaF) [24]. When mentioned, iopromide was the most commonly utilised contrast agent mentioned in seven studies [16,17,18,22,27,34,35]; other contrast agents used were iopamidol [23,31] and iohexol [30]. Where available, the studies reported using tube voltages between 80 kV to 120 kV. The tube current ranged from 30 mA [19] to 800 mA [23]. Most studies reported using a slice thickness within the range of 0.5 mm to 0.75 mm [16,17,18,20,29,30,31,32], although You et al. opted for 0.9 mm [34]. Similarly, with the exception of You et al. [34], the slice interval was set at 0.25 mm [20,21,22,23,24,25,26,27,28,29,30], 0.5 mm [17,18,32] or 0.625 mm [16,31] in the remaining studies.

**Table 2:** Imaging and radiomics methodology.

Study	Modality	Radiomics Architecture	Segmentation and Processing	Performance Evaluation
**Carotid studies**				
**Chen et al.** [14]	CT angiography	**Adherence to radiomics guidelines:** nil **Feature extraction software:** 3D Slicer (https://www.slicer.org/, accessed on 27 August 2024)	**Segmentation:** manual segmentation of the coronary plaque and semiautomated segmentation of the PVAT using 3D Slicer (https://www.slicer.org/, accessed on 27 August 2024) **Features extracted:** shape, first order, GLCM, GLDM, GLSZM, GLRLM and NGTDM **Machine learning techniques:** SVM	**Performance assessment:** AUC from the ROC, accuracy, sensitivity, specificity, PPV, and NPV **Internal validation:** dataset split into training set (n = 100) and validation set (n = 44). Tenfold cross validation **No external validation**
**Cilla et al.** [15]	CT angiography	**Adherence to radiomics guidelines:** radiomic feature extraction performed in accordance with IBSI **Feature extraction software:** Moddicom (radiomics software package for R, https://github.com/kbolab/moddicom, accessed on 27 August 2024)	**Segmentation:** manual segmentation **Features extracted:** first order, shape, GLCM, GLRLM, GLSZM, NGTDM and GLDM **Machine learning techniques:** logistic regression, SVM, CART	**Performance assessment:** AUC from the ROC, AUC, class-specific accuracy (proportion of both true positive and true negatives amongst all cases), PPV, sensitivity and *F*-measure **Internal validation:** fivefold cross validation applied to each machine learning model **No external validation**
**Ebrahimian et al.** [26]	Dual-energy CT angiography	**Adherence to radiomics guidelines:** nil **Feature extraction software:** PyRadiomics integrated into Dual-Energy Tumour Analysis prototype software (eXamine, Siemens Healthineers, Forcheim, Germany)	**Segmentation:** automated segmentation using Dual-Energy Tumour Analysis prototype software (eXamine, Siemens Healthineers, Forcheim, Germany) **Features extracted:** shape, first-order, GLCM, NGTDM, GLSZM, GLRLM, GLDM, and higher-order features **Machine learning techniques:** multinomial logistic regression	**Performance assessment:** AUC from the ROC **Internal validation:** DNM **No external validation**
**Kafouris et al.** [36]	PET/CT using 0.14 mCi/kg ^18^F-FDG	**Adherence to radiomics guidelines:** features extracted according to IBSI guidelines **Feature extraction software:** in-house software based on Matlab platform (Version 9.3, Matlab R2017b, Natick, MA, USA)	**Segmentation:** manual segmentation around the carotid artery wall **Features extracted:** first order, GLCM, GLRLM, GLSZM and NGTDM **Machine learning techniques:** univariate logistic regression	**Performance assessment:** AUC from the ROC **Internal validation:** bootstrapping generating 200 bootstrap samples **No external validation**
**Liu et al.** [37]	CT angiography	**Adherence to radiomics guidelines:** nil **Feature extraction software:** Radcloud platform (Huiying Medical Technology, Beijing, China)	**Segmentation:** manual segmentation of the coronary plaque using ITK-SNAP software (version 3.7, http://www.itksnap.org/, accessed on 27 August 2024) **Features extracted:** shape, first order, GLDM, GLRLM, GLCM, GLSZM and NGTDM **Machine learning techniques:** LASSO used to construct a ‘radiomics score’	**Performance assessment:** AUC from the ROC **Internal validation:** dataset split into training set (n = 135) and validation set (n = 58) **External validation using 87 patients**
**Nie et al.** [38]	CT angiography	**Adherence to radiomics guidelines:** nil **Feature extraction software:** Shukun AI Scientific Research Platform (Shukun Technology, Beijing, China)	**Segmentation:** automated segmentation of the PVAT using Perivascular Fat Analysis Software (Shukun Technology, Beijing, China) **Features extracted:** first order, shape, GLCM, GLDM, GLRLM, GLSZM and NGTDM **Machine learning techniques:** Bagging DecisionTree, XGBoost, random forest, SVM and quadratic discriminant analysis	**Performance assessment:** AUC from the ROC **Internal validation:** dataset split into training set (n = 163) and test set (n = 40) **No external validation**
**Le et al.** [39]	CT angiography	**Adherence to radiomics guidelines:** nil **Feature extraction software:** PyRadiomics (version 3.0, https://pyradiomics.readthedocs.io/, accessed on 27 August 2024)	**Segmentation:** manual segmentation using TexRad (Feedback Medical Ltd., London, UK) **Features extracted:** first order, GLCM, GLRLM, GLSZM, GLDM, and NGTDM **Machine learning techniques:** decision tree, random forest, LASSO, Elastic Net regression (weight for *L*1 and *L*2 penalties = 0.5), neural network, and XGBoost	**Performance assessment:** AUC from the ROC **Internal validation:** fivefold cross validation **No external validation**
**Shan et al.** [40]	CT angiography	**Adherence to radiomics guidelines:** nil **Feature extraction software:** PyRadiomics integrated into Python	**Segmentation:** semi-automated segmentation using 3D Slicer **Features extracted:** shape, first order, GLDM, GLRLM, GLCM, GLSZM and NGTDM **Machine learning techniques:** logistic regression, SVM, random forest, light gradient boosting machine, AdaBoost, XGBoost, and multi-layer perception	**Performance assessment:** AUC from the ROC **Internal validation:** dataset split into training set and validation set in a ratio of 7:3 **No external validation**
**Shi et al.** [41]	CT angiography	**Adherence to radiomics guidelines:** nil **Feature extraction software:** The Deepwise Multimodal Research Platform (version 2.0, Beijing Deepwise & League of PHD Technology Co. Ltd, Beijing, China)	**Segmentation:** manual segmentation of the coronary plaque using The Deepwise Multimodal Research Platform (version 2.0, Beijing Deepwise & League of PHD Technology Co. Ltd, Beijing, China) **Features extracted:** shape, first order, GLDM, GLRLM, GLCM, GLSZM and NGTDM **Machine learning techniques:** analysis of variance F-value, mutual information and linear models penalised with the L1 norm	**Performance assessment:** AUC from the ROC, calibration, and decision curve analyses **Internal validation:** fivefold cross validation applied to each machine learning model **No external validation**
**Xia et al.** [42]	CT angiography	**Adherence to radiomics guidelines:** nil **Feature extraction software:** PyRadiomics (version 2.4) integrated into Python	**Segmentation:** manual segmentation of the coronary plaque using 3D Slicer (version 4.11) **Features extracted:** shape, first order, GLCM, GLSZM, GLRLM, NGTDM and GLDM **Machine learning techniques:** random forest, XGBoost, logistic regression, SVM and k-nearest neighbour	**Performance assessment:** predictive value of the model assessed using AUC from the ROC **Internal validation:** dataset split into training set (n = 165) and validation set (n = 66). Fivefold cross validation used on the training set **No external validation**
**Coronary studies**				
**Chen et al.** [16]	CT coronary angiography	**Adherence to radiomics guidelines:** nil **Feature extraction software:** Perivascular Fat Analysis Tool	**Segmentation:** semi-automated segmentation of the PCAT using Perivascular Fat Analysis Tool **Features extracted:** shape, first order, GLDM, GLCM, GLRLM, GLSZM and NGTDM **Machine learning techniques:** multivariate logistic regression used to construct a ‘radiomics score’	**Performance assessment:** AUC from the ROC **Internal validation:** dataset split into training set (n = 108) and validation set (n = 47). Fivefold cross validation performed **No external validation**
**Chen et al.** [17]	CT coronary angiography	**Adherence to radiomics guidelines:** features extracted according to IBSI guidelines **Feature extraction software:** Radiomics, Syngo.Via FRONTIER (version 1.2.1, Siemens Healthineers, Forcheim, Germany)	**Segmentation:** manual segmentation using Radiomics, Syngo.Via FRONTIER (version 1.2.1, Siemens Healthineers, Forcheim, Germany) **Features extracted:** shape, first order, GLCM, GLSZM, GLRLM, GLDM and NGTDM **Machine learning techniques:** multivariable logistic regression and XGBoost used to construct the algorithm	**Performance assessment:** predictive value of the model assessed using AUC from the ROC **Internal validation:** dataset split into training set and validation set in a ratio of 7:3. Fivefold cross validation used on the training set (n = 137) **External validation using 159 patients**
**Feng et al.** [18]	CT coronary angiography	**Adherence to radiomics guidelines:** nil **Feature extraction software:** Radiomics, Syngo.Via FRONTIER (version 1.3.0)	**Segmentation:** semi-automated segmentation of the plaque using Coronary Plaque Analysis Syngo.Via Frontier (version 5.0.2, Siemens Healthineers, Forcheim, Germany) **Features extracted:** shape, first order and texture **Machine learning techniques:** random forest model and logistic regression used to construct the radiomics model	**Performance assessment:** AUC from the ROC, sensitivity, specificity, and accuracy **Internal validation:** dataset split into training set (n = 280) and validation set (n =120) **No external validation**
**Homayounieh et al.** [19]	CT coronary angiography	**Adherence to radiomics guidelines:** nil **Feature extraction software:** Radiomics, Syngo.Via FRONTIER	**Segmentation:** automated segmentation using Radiomics, Syngo.Via FRONTIER **Features extracted:** shape, first order, GLCM, GLRLM, GLSZM, NGTDM and GLDM **Machine learning techniques:** multiple logistic regression and kernel Fisher discriminant analysis	**Performance assessment:** AUC from the ROC **Internal validation:** nil **No external validation**
**Hou et al.** [20]	CT coronary angiography	**Adherence to radiomics guidelines:** nil **Feature extraction software:** DNM	**Segmentation:** semi-automated segmentation of the PCAT **Features extracted:** first order, GLCM, GLRLM, GLSZM, GLDM and NGTDM **Machine learning techniques:** SVM, k-nearest neighbour, Light GBM, and random forest	**Performance assessment:** AUC from the ROC **Internal validation:** dataset split into training set (n = 123) and validation set (n = 54). Tenfold cross validation used on the training set **No external validation**
**Hu et al.** [21]	CT coronary angiography	**Adherence to radiomics guidelines:** nil **Feature extraction software:** PyRadiomics library integrated into an unknown software	**Segmentation:** manual segmentation using ITK-SNAP software (version 3.6.0) **Features extracted:** first order, shape, texture, higher order **Machine learning techniques:** logistic regression	**Performance assessment:** AUC from the ROC, sensitivity, specificity, PPV, NPV, positive likelihood ratio, negative likelihood ratio **Internal validation:** dataset split into training set (n = 88) and validation set (n = 31) **No external validation**
**Jing et al.** [22]	CT coronary angiography	**Adherence to radiomics guidelines:** nil **Feature extraction software:** PyRadiomics library integrated into Pericoronary Adipose Tissue Analysis Software (Shukun Technology, Beijing, China)	**Segmentation:** automated segmentation using CoronaryDoc software (Shukun Technology, Beijing, China) **Features extracted:** first order and texture features **Machine learning techniques:** SVM, ridge regression classifier and logistic regression	**Performance assessment:** AUC from the ROC, accuracy, specificity, sensitivity, PPV, and NPVs **Internal validation:** dataset split into training set and validation set at a ratio of 2:1. Fivefold cross validation performed **No external validation**
**Kim et al.** [23]	CT coronary angiography	**Adherence to radiomics guidelines:** features extracted according to IBSI guidelines **Feature extraction software:** PyRadiomics integrated into Python	**Segmentation:** semi-automated segmentation of the PCAT using in-house Python software **Features extracted:** shape, first order, GLCM, GLDM, GLRLM, GLSZM and NGTDM **Machine learning techniques:** multivariate logistic regression	**Performance assessment:** predictive value of the model assessed using AUC from the ROC **Internal validation:** stratified threefold cross validation performed **No external validation**
**Kwiecinski et al.** [24]	PET/CT performed using 250 MBq ^18^F-NaF	**Adherence to radiomics guidelines:** nil **Feature extraction software:** Radiomics Image Analysis (version 1.4.2, https://github.com/neuroconductor/RIA, accessed on 27 August 2024) on R	**Segmentation:** automated segmentation of the PET/CT using coronary microcalcification activity. Semi-automated segmentation of the plaques from the CTCA using Autoplaque (version 2.5, Cedars-Sinai Medical Center, Los Angeles, CA, USA) **Features extracted:** DNM type of features extracted **Machine learning techniques:** univariable and multivariable logistic regression, linear regression and random forest	**Performance assessment:** nil **Internal validation:** DNM **No external validation**
**Lee et al.** [25]	CT coronary angiography	**Adherence to radiomics guidelines:** nil **Feature extraction software:** PyRadiomics integrated into Python	**Segmentation:** semi-automated segmentation of the coronary plaque using QAngioCT Research Edition (version 2.1.9.1, Medis Medical Imaging, Leiden, Netherlands) **Features extracted:** first order, GLCM, GLRLM, GLSZM, GLDM and NGTDM **Machine learning techniques:** multivariable Cox regression model	**Performance assessment:** AUC from the ROC **Internal validation:** dataset split into training set and validation set in a ratio of 8:2 **No external validation**
**Li et al.** [27]	CT coronary angiography	**Adherence to radiomics guidelines:** nil **Feature extraction software:** PyRadiomics integrated into Python	**Segmentation:** manual segmentation of the coronary plaque **Features extracted:** shape, first order, GLCM, GLDM, GLRLM, GLSZM and NGTDM **Machine learning techniques:** Naïve Bayes, decision tree, random forest, gradient boosting decision tree, SVM, multilayer perceptron, logistic regression, and k-nearest neighbours	**Performance assessment:** AUC from the ROC **Internal validation:** dataset split into training set (n = 36) and validation set (n = 8). Fivefold cross validation performed on the training set **No external validation**
**Li et al.** [28]	CT coronary angiography	**Adherence to radiomics guidelines:** nil **Feature extraction software:** PyRadiomics integrated into Research Portal (version 1.1, United Imaging Intelligence Co. Ltd., Shanghai, China)	**Segmentation:** automated segmentation of the coronary plaque using Research Portal (version 1.1) **Features extracted:** shape, first order, GLCM, GLRLM, GLSZM, NGTDM and GLDM **Machine learning techniques:** DNM	**Performance assessment:** AUC from the ROC **Internal validation:** dataset split into training set and validation set in a ratio of 8:2. Fivefold cross validation performed **External validation using 50 patients**
**Lin et al.** [29]	CT coronary angiography	**Adherence to radiomics guidelines:** nil **Feature extraction software:** Radiomics Image Analysis software package (version 1.4.1) on R	**Segmentation:** automated segmentation of the PCAT using Autoplaque software (version 2.5) **Features extracted:** shape, first order features, GLCM and GLRLM **Machine learning techniques:** XGBoost	**Performance assessment:** AUC from the ROC **Internal validation:** tenfold cross validation **No external validation**
**Lin et al.** [30]	CT coronary angiography	**Adherence to radiomics guidelines:** nil **Feature extraction software:** Radiomics Image Analysis software package (version 1.4.2) on R	**Segmentation:** semi-automated segmentation of the coronary plaque using Autoplaque (version 2.5) **Features extracted:** shape, first order, GLCM and GLRLM **Machine learning techniques:** XGBoost	**Performance assessment:** AUC from the ROC **Internal validation:** tenfold cross validation **External validation on 19 patients**
**Oikonomou et al.** [31] **(study 2 and 3)**	CT coronary angiography	**Adherence to radiomics guidelines:** nil **Feature extraction software:** PyRadiomics integrated into 3D Slicer	**Segmentation:** manual segmentation of the PVAT **Features extracted:** shape, first order, GLCM, GLDM, GLRLM, GLSZM, NGTDM and higher order **Machine learning techniques:** random forest	**Performance assessment:** predictive value of the model assessed using AUC from ROC **Internal validation:** dataset split into training set and validation set in a ratio of 4:1. Fivefold cross validation performed **External validation performed on the validation dataset**
**Si et al.** [32]	CT coronary angiography	**Adherence to radiomics guidelines:** nil **Feature extraction software:** Research Portal (version 1.1)	**Segmentation:** automated segmentation using the VB-net model **Features extracted:** shape, first order, GLCM, GLRLM, GLSZM, GLDM and NGTDM **Machine learning techniques:** logistic regression	**Performance assessment:** AUC from the ROC **Internal validation:** dataset split into training set and validation set in a ratio of 7:3. Fivefold cross validation performed **No external validation**
**Wen et al.** [33]	CT coronary angiography	**Adherence to radiomics guidelines:** nil **Feature extraction software:** PyRadiomics integrated into 3D Slicer (version 4.10.2)	**Segmentation:** manual segmentation of the PCAT using 3D slicer **Features extracted:** first order, GLCM, GLRLM, GLSZM, GLDM and higher order **Machine learning techniques:** logistic regression, decision tree and SVM	**Performance assessment:** AUC from the ROC **Internal validation:** dataset split into training set and validation set in a ratio of 4:1 **No external validation**
**You et al.** [34]	CT coronary angiography	**Adherence to radiomics guidelines:** nil **Feature extraction software:** Artificial Intelligence Kit (GE Healthcare, Chicago, IL, USA)	**Segmentation:** semi-automated segmentation of the epicardial adipose tissue using EATseg software (https://github.com/MountainAndMorning/EATSeg, accessed on 27 August 2024) and 3D slicer (version 4.11) **Processing:** nil **Features extracted:** first order, GLCM, GLSZM, GLRLM, NGTDM and GLDM **Machine learning techniques:** logistic regression	**Performance assessment:** AUC from the ROC **Internal validation:** dataset split into training set and validation set in a ratio of 7:3 **No external validation**
**Yu et al.** [35]	CT coronary angiography	**Adherence to radiomics guidelines:** nil **Feature extraction software:** PyRadiomics integrated into an in-house software	**Segmentation:** automated segmentation using CoronaryDoc, FAI Analysis Tool (version 5.1.2, Shukun Technology, Beijing, China) **Features extracted:** first order, GLCM, GLSZM, GLRLM, NGTDM and GLDM **Machine learning techniques:** SVM	**Performance assessment:** AUC from the ROC **Internal validation:** dataset split into training set and validation set in a ratio of 2:1. Fivefold cross validation performed applied to training set **No external validation**

Abbreviations: CT = computed tomography, PVAT = peri-vascular adipose tissue, GLCM = grey-level co-occurrence matrix, GLDM = grey-level dependence matrix, GLSZM = grey-level size zone matrix, GLRLM = grey-level run length matrix, NGTDM = neighbouring grey tone difference matrix, SVM = support vector machine, AUC = area under curve, ROC = receiver operating characteristic, PPV = positive predictive value, NPV = negative predictive value, IBSI = Image Biomarker Standardisation Initiative, CART = classification and regression tree, DNM = does not mention, PET = positron emission tomography, mCi = millicurie, kg = kilogram, ^18^F-FDG = [¹⁸F]Fluorodeoxyglucose, LASSO = least absolute shrinkage and selection operator, PCAT = peri-coronary adipose tissue,. CTCA = computed tomography coronary angiography, MBq = megabecquerel,^18^F-NaF = [¹⁸F]sodium fluoride.

### 3.4. Segmentation

The region of interest (ROI) varied amongst the carotid artery studies (Supplementary Table S4). Eight reports [15,26,36,37,39,40,41,42] focused on segmentation of the carotid plaque, one study [38] contoured the peri-vascular adipose tissue, and a further study [14] extracted data from both the plaque and the peri-vascular adipose tissue. In the coronary artery studies, the plaque was the ROI in nine studies [17,18,19,21,24,25,27,28,30], whilst nine other evaluations [16,20,22,23,29,31,32,33] focused on peri-coronary adipose tissue, and a single group [35] extracted data from both the peri-coronary and the epicardial adipose tissue. 

A variety of approaches to ROI definition were adopted, including manual [15,17,21,27,31,33,36,37,39,41,42], semi-automated [16,18,23,25,30,34,40] or automated segmentation [19,22,24,26,28,29,32,35,38]. Moreover, in some studies, different ROIs underwent combinations of segmentation, including manual with semi-automated segmentation [14] or semi-automated with automated segmentation [20]. Where manual segmentation was performed, this process was undertaken by one individual in three studies [27,41,42] or two individuals in eight studies [14,15,16,18,21,34,37,40]. The most commonly used software was 3D Slicer. Further information on the segmentation methodology is outlined in Table 2 and Supplementary Table S3.

### 3.5. Processing

An array of image processing methods were used (Supplementary Table S3). In some studies, the voxels were discretised into fixed bin widths of 25 HU [14,17,26,33] or into a specific number of bins, such as 8 [23,29,30], 16 [23,29,30,31], 32 [23,29,30] or 64 [36]. Other studies reported the resampling of voxels to 1 × 1 × 1 mm [14,17,20]. Various filtration methods were used, including Wavelet transform [14,16,18,27,28,40], Laplacian of Gaussian [14,16,18,27,40], exponential filter [27,40], gradient filter [40], Laplacian sharpening filter [28] and non-linear transformation filter [16,18]. Some studies did not report any processing steps [15,19,21,22,24,25,35,37,38,41,42].

### 3.6. Radiomic Feature Extraction

**Figure 3:**
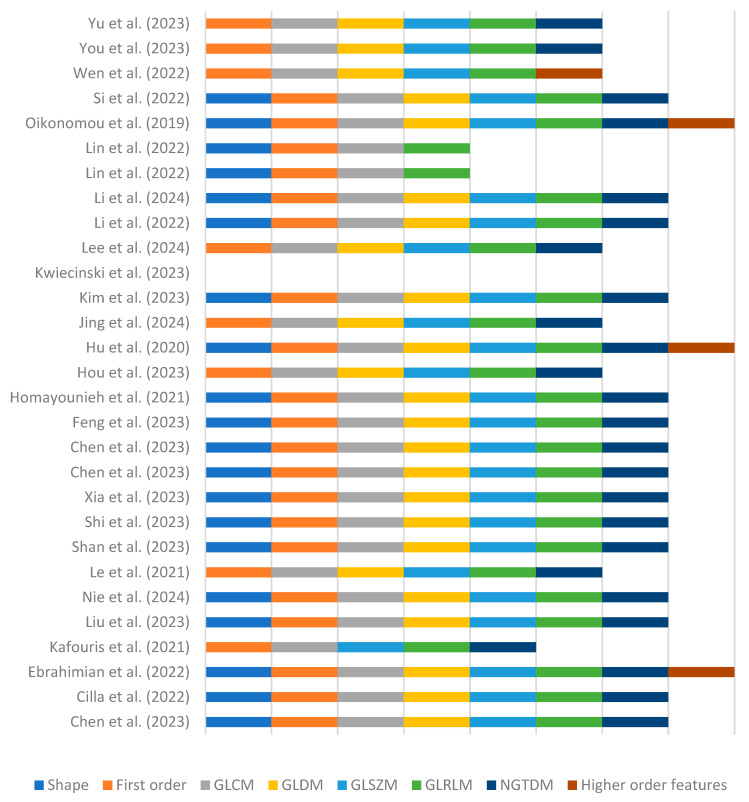
Stacked plot diagram highlighting the extracted radiomic feature classes from the different studies [14,15,16,17,18,19,20,21,22,23,24,25,26,27,28,29,30,31,32,33,34,35,36,37,38,39,40,41,42]. Abbreviations: GLCM = grey-level cooccurrence matrix, GLDM = grey-level dependence matrix, GLSZM = grey-level size zone matrix, GLRLM = grey-level run length matrix and NGTDM = neighbouring grey tone difference matrix.

A minority of studies [15,17,23,36] reported adherence to published radiomics guidelines; this involved feature extraction in accordance with the Image Biomarker Standardization Initiative [48]. Most commonly, feature extraction was performed using the PyRadiomics package integrated into various software [21,22,23,25,26,27,35,39,40,42]. Alternatively, some authors reported using the R platform [15,24,29,30], 3D Slicer [14,31,33] or an in-house software [36]. The types of features extracted are shown in Figure 3.

### 3.7. Dimensionality Reduction and Feature Selection

**Figure 4:**
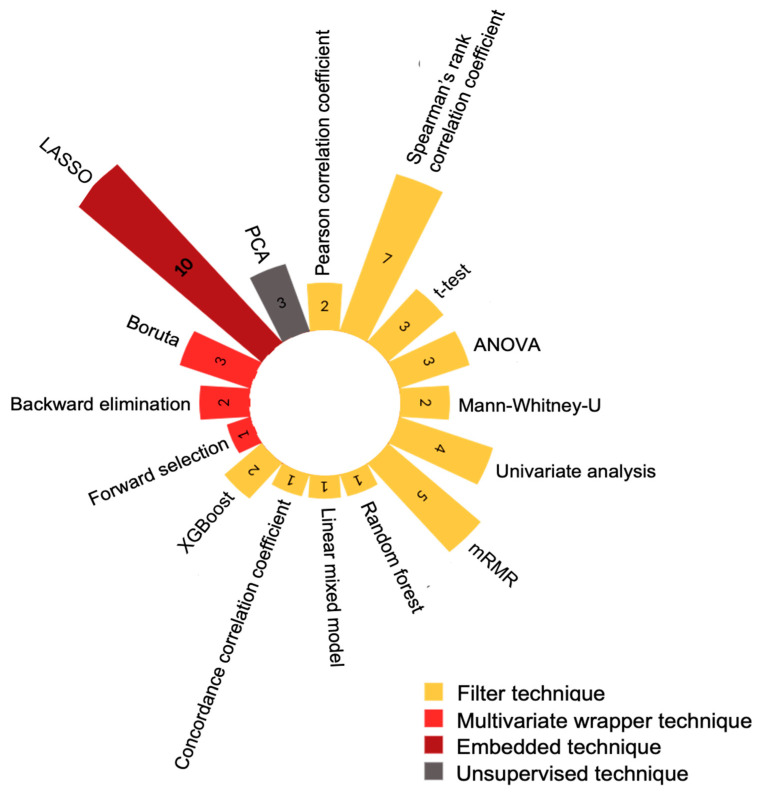
Polar graph demonstrating the different feature selection techniques used. Abbreviations: ANOVA = analysis of variance, mRMR = minimum redundancy maximum relevance, XGBoost = extreme gradient boosting, LASSO = least absolute shrinkage and selection operator and PCA = principal component analysis.

In 11 studies [14,16,17,20,21,31,33,35,37,39,40,41], intraclass correlation was used to assess the reproducibility of the image segmentation technique between different clinicians. Studies used an intraclass correlation threshold of 0.75 [14,20,21,33,37], 0.8 [17,41], 0.85 [35] or 0.9 [16,31,39,40] when selecting radiomic features. A variety of dimensionality reduction and feature selection techniques were employed as shown in Figure 4. Generally, a combination of statistical methods were used; however, in five studies [18,27,29,30,42], a single method was selected (Supplementary Table S3). Additionally, Houmayounieh et al. [19] did not specify the statistical method utilised in their study. 

### 3.8. Machine Learning Methods

**Figure 5:**
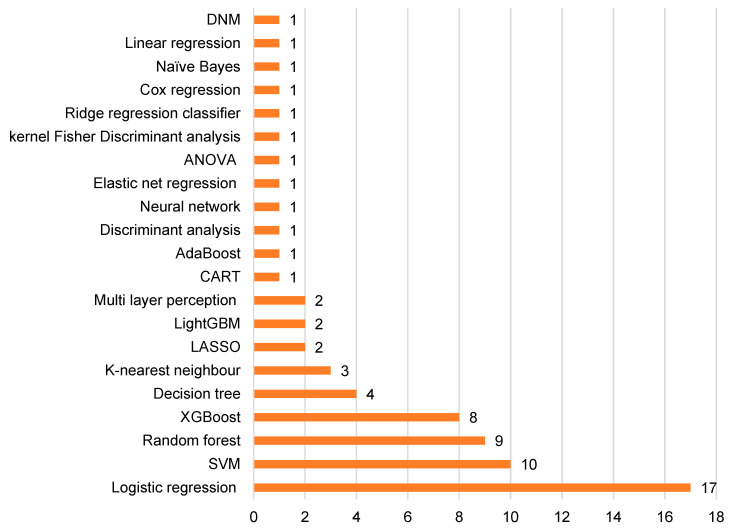
Bar chart showing different machine learning methods applied. Abbreviations: DNM = does not mention, ANOVA = analysis of variance, CART = classification and regression tree analysis, LightGBM = light gradient boosting machine, LASSO = least absolute shrinkage and selection operator, XGBoost = extreme gradient boosting and SVM = support vector machine.

In total, 21 different machine learning methods were used as illustrated in Figure 5. The median number of machine learning methods used per study was one. When stratified by disease type, the median number of machine learning methods used were two in carotid studies and one in coronary studies. 

In the carotid studies, the outcomes of interest included differentiating between symptomatic and asymptomatic lesions [14,37,38,39,41], distinguishing between vulnerable and non-vulnerable lesions [15,36,40], predicting surgical outcomes [26] and predicting complications such as a transient ischaemic attack arising from lesions [42] (Supplementary Table S4). Symptomatic plaques referred to sequalae such as stroke or transient ischaemic attack arising from atherosclerotic disease. Vulnerable plaques were defined using histological analysis [36], immunohistological analysis [36] or invasive angiography [40].

Machine learning methods in the coronary studies were used to predict structural changes to arterial plaques such as rapid progression [17,18] stenosis [19,25,31,33,35] or complete obstruction [16], whilst, in other studies, functional changes such as myocardial ischaemia were predicted (Supplementary Table S4) [20,21]. Alternatively, modelling was utilised to predict clinical outcomes such as the MESA CHD risk [19], major adverse cardiovascular events (MACEs) [21,34] or acute coronary syndrome (ACS) [22,24]. Other studies focused on using radiomic analysis to differentiate rather than predict. This included differentiating between types of structural changes, such as occluded and non-occluded arteries [28] (validated using invasive coronary angiography) or culprit and non-culprit lesions. [30] Alternatively, clinical sequalae differences in the severity of ACS [29] or between MACE and non-MACE cases [31] were explored. Two studies utilised radiomic analysis to identify vulnerable plaques [23,27] that were validated using optical coherence tomography [23] or histological analysis [27].

With the exception of four studies [15,23,24,40], a comparator model was used to assess the performance of the radiomics models (Supplementary Table S4). Comparator models incorporated conventional CT features only [13,15,16,17,19,21,25,26,27,29,30,31,32,34,36,37,38], conventional PET/CT features only [36], clinical features only [19,34,42] or a combination of conventional CT and clinical features [25,29,34,41]. Additionally, some studies evaluated the performance of models that combined conventional imaging features with radiomic features [14,17,18,25,28,29,32,33,37,38,40,41,42] or clinical features with radiomic features [19,29,34].

### 3.9. Performance Evaluation and Validation

**Figure 6:**
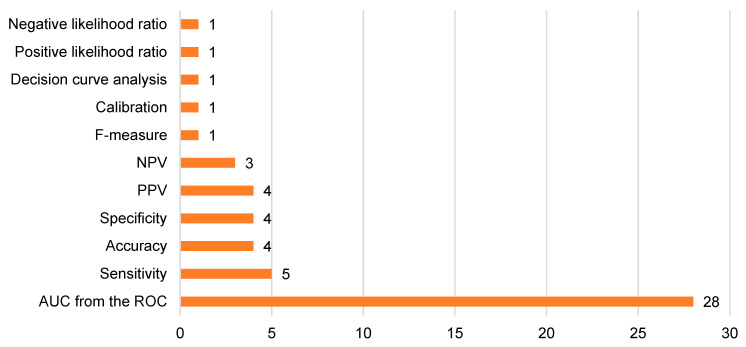
Bar chart illustrating different model performance assessment methods. Abbrevations: NPV = negative predictive value, PPV = positive predictive value and AUC from the ROC = area under the curve from the receiver operator characteristic curve.

The majority of studies [14,15,16,17,18,19,20,21,22,23,25,26,27,28,29,30,31,32,33,34,35,36,37,38,39,40,41,42] used area under the curve from the receiver operator characteristic curve to assess the performance of machine learning methods (Figure 6). One study [24] did not describe their method of performance assessment. Nineteen studies [14,16,17,18,20,21,22,25,27,28,31,32,33,34,35,37,38,40,42] split data into training sets and validation sets. Three- [23], five- [15,16,17,22,27,28,31,32,35,39,41,42] or ten-fold [14,20,29,30] cross validation was performed in 1, 12 and 4 studies, respectively. Alternatively, Kafouris et al. [36] performed bootstrapping to validate their model. Moreover, in five studies [17,28,30,31,37], external validation was performed, comprising 315 patients.

## 4. Discussion

This review highlights the increasing frequency of publications exploring radiomics in the cardiovascular imaging domain. This corroborates with findings from Pinto dos Santos et al. [49], who observed an exponential increase in publications pertaining to radiomics from 2012 to 2019, predominantly in the oncology setting. To explore the clinical applicability of radiomic analysis in cardiovascular disease, all studies included in this scoping review applied radiomic analysis to predict clinical outcomes such as death, restenosis, stroke or myocardial infarction. This demonstrated that clinically relevant questions were being explored, most research was single-centre and retrospective observational studies that lacked adherence to published guidelines or external validation of the results limited the reproducibility of their findings. Consequently, many of the studies served as feasibility/proof of concept works.

Approximately four-fifths of the patients were evaluated in coronary artery studies. This disparity is likely due to differences in the approach to imaging carotid disease and coronary disease. Duplex ultrasound is the modality of choice for investigating carotid disease; however, the inclusion of this technique was outside the remit of this review. In contrast, CTA of the carotid arteries is recommended as a second-line investigation by the ESVS [47]; hence, fewer patients would have been routinely investigated using this modality. CT coronary angiography (CTCA) is recommended as the primary imaging modality for coronary disease, which facilitates radiomic analysis using large datasets of routinely available imaging. Additionally, in the field of coronary disease, imaging data were also derived from large multi-centre clinical trials, such as the SCOT-HEART trial [31].

Voxel intensity discretisation reduces the range of intensity values to a computationally practical number to facilitate radiomic analysis. This is most commonly achieved through two different methods [5]. Firstly, voxel intensity values can be organised into a fixed number of bins, most commonly into 2*^N^* bins (with *N* ranging 3 to 8), as observed in five studies [23,29,30,31,36]. Alternatively, intensities can be discretised into equally sized bins with a fixed bin width that enables the comparison of different images, as the bins with the comparative ranges will represent the same data intervals [5]. In this scoping review, in five studies [14,17,26,33], a fixed bin width of 25 HU was used. Setting an optimal bin number can be challenging, as having too few can cause features to be averaged out between the bins whilst having too many bins can preclude the identification of features from background noise. Overall, the impact of discretisation on feature reduction is equivocal: Shafiq-ul-Hassan et al. [50] found that 44 out of 51 radiomic features were dependent on the grey-level discretisation, whereas Larue et al. [51] noted that the stability of radiomic features was not significantly influenced by choice of bin widths.

This scoping review included a range of imaging modalities to reflect contemporary clinical practice. For instance, CTA is a second-line imaging modality to investigate carotid disease as stipulated by the ESVS [47] and was used in nine studies. Similarly, CTCA is recommended for the identification of coronary artery disease by the European Society of Cardiology [4] and was utilised in 18 studies. This highlights the potential to apply radiomic analysis to imaging datasets from real world practice to support large-scale research. Additionally, alternative imaging modalities such as PET/CT were also included to reflect novel research directions.

There was variability in scanning parameters between different studies and a lack of standardisation in the acquisition protocols adopted by different imaging centres. This is an important consideration, as evidence has demonstrated that factors such as tube current [52], slice thickness [53] and contrast enhancement [54] impact what radiomic features are extracted from images. Consequently, the repeatability of radiomics studies is contingent on consistency in image acquisition and reconstruction protocols. To address this, there are various options, including adhering to published image acquisition guidance such as the British Society of Cardiovascular Imaging Standards of practice of CTCA [55] or employing a dummy object consisting of various densities to adjust scanning parameters and standardise protocols between centres [56].

The clinical significance of the peri-vascular adipose tissue has been highlighted through research into conventional CT features. For instance, the literature has demonstrated that peri-coronary adipose tissue is associated with coronary plaques [57] and an increased risk of death [58], whilst attenuation has been used to differentiate between flow-limiting and non-flow-limiting lesions [59]. Nonetheless, there is a paucity of evidence summarising published research on the radiomic analysis of the peri-vascular adipose tissue; this scoping review has demonstrated that there are numerous published studies both in carotid disease [38] and coronary disease [16,22,33,35].

There was diversity in the segmentation methods utilised in different studies. The choice of segmentation technique can be dependent on operator experience and the availability of software for automation of this process. Semi-automated or manual segmentation is susceptible to observer bias, labour-intensive and time-consuming [5]. Contrarily, automated segmentation is faster and reduces inter- and intraobserver variability. Nonetheless, the published evidence on the superiority of automated segmentation is equivocal. Gresser et al. [60] observed a higher predictive ability of a lymph node manual segmentation model for detecting bladder cancer when compared to an automated model, whilst another study [61] investigating radiomic features of hypopharyngeal cancer on MRI found that automated segmentation models based on the DeepLab V3+ and U-Net architectures performed similar to manual segmentation. With respect to automated segmentation, this review identified that a variety of different software based on convolutional neural networks such as U-Net, Rb-Net and V-Net were employed.

### Limitations and Areas for Further Research

Several limitations were identified in the existing literature. An inherent shortcoming of radiomics is a lack of generalisability of findings across different settings or population groups. Given that the fundamentals of radiomic features pertain to the distribution and relationship between different parts of an image, this is contingent on how images are acquired, segmented and analysed. A lack of standardised imaging acquisition protocols between different radiological centres introduce variations in the acquisition and reconstruction of images, leading to changes in the images that may fail to reflect the underlying variation in pathology. Similarly, in the case of manual segmentation, different readers may interpret images differently, resulting in the loss of important data. In the case of automated segmentation, algorithms that perform complex computations can be derailed by background noise. This can be addressed by reporting measures of error, using standardised imaging acquisition protocols and reporting the inter-operator variability for image segmentation.

Another limitation was heterogeneity in reporting between the studies. For example, some studies reported the techniques used for feature selection without expanding into detail on what criteria were used to select or discard features. This is an important consideration, as the selection of features with high repeatability and reproducibility is necessary to reduce the risk of false discovery (type 1 error) [62]. To ameliorate this, the use of a quality assessment tool such as CheckList for EvaluAtion of Radiomics research (CLEAR) [63], METRICS or the Radiomics Quality Score [64] is recommended to foster transparent high-quality reporting. 

The translatability of the research included in this review into clinical practice is limited. Most of the included research constituted retrospective single-centre studies, and only five studies performed validation of their methodology using external datasets. To address this limitation, higher-level research such as multi-centre prospective randomised controlled trials is necessary, consisting of large sample sizes to improve the predictive power of the machine learning algorithms. Another possible approach is to utilise big data through shared datasets obtained from routine clinical data. This is advantageous, as it increases the study sample size, thus improving the predictive ability of machine learning models and accounting for variations in disease processes between different individuals and capturing temporal changes in imaging technology [7]. Nonetheless, such an approach requires investment into hardware infrastructure and is contingent on legal and ethical regulations.

As outlined by Munn et al., the indications for systematic reviews and scoping reviews differ [65]. The former type of evidence synthesis is generally utilised to evaluate all the available evidence for a well-defined clinical question and establish the effectiveness or appropriateness of interventions that address that question. In contrast, scoping reviews are used to identify the key concepts and methodology related to a broad clinical question. In the case of this scoping review, the effectiveness of the radiomic techniques using area under the curve (AUC) values or receiver operating characteristic (ROC) values was not explored, as this level of examination is generally undertaken in systematic reviews. Nonetheless, this could be covered in a subsequent systematic review.

Finally, there is a paucity of evidence on economic evaluations in the field of radiomics. In the oncology setting, Di Pilla et al. [66] conducted an economic evaluation of a screening program for the identification of BRCA 1/2 carriers and demonstrated an incremental cost-effectiveness ratio of between EUR 653 and EUR 3800 for a radio-genomic model based on ultrasound imaging. In theory, radiomics could result in health benefits by extracting additional data from clinical images used to inform clinical management at a low cost. For instance, in the field of cardiovascular medicine, radiomics could be used to tailor imaging surveillance regimes according to the predicted risk, resulting in cost savings as compared to a blanket screening programme. Moreover, in high-risk patients, more aggressive treatment could be initiated to prevent potential complications. 

## 5. Conclusions

This review highlights published research on radiomic analysis of the coronary and carotid arteries using CT and PET/CT imaging modalities. Much of the evidence is single-centre, retrospective observational studies with limited generalisability or repeatability. There is significant heterogeneity in the image acquisition protocols, segmentation techniques, processing and analysis between the studies. To improve the clinical applicability of radiomics, there is a need for the implementation of standardised imaging acquisition guidelines, adherence to published reporting guidelines, quality analysis of the results and economic evaluation of the costs and benefits. These changes can facilitate the expansion of radiomic analysis into smaller and peripheral imaging centres, thus creating more robust consolidated techniques.

## Figures and Tables

**Table 1:** Study characteristics. Continuous variables displayed using mean ± standard deviation or median (interquartile range).

Study	Patient Demographics	Age (Years)	Eligibility Criteria	Comorbidities (Number of Patients)
**Carotid studies**				
**Chen et al.** [14] Single-centre study	Overall: 144 Male: 110	70.9 ± 9.1	**Inclusion criteria:** diagnosis of extracranial carotid stenosis between 30–99% on CTA images, sufficient information to ascertain cerebral ischemia symptoms in the medical records, and adequate information regarding vascular risk factors in the medical records **Exclusion criteria:** cardiogenic stroke, simultaneous bilateral anterior circulation events, complications of radiation therapy and vasculitis, stroke involving the posterior circulation only, inadequate image quality	Hypertension: 111 Hyperlipidaemia: 69 Smoker: 65 Diabetes mellitus: 52 CAD: 40
**Cilla et al.** [15] Single-centre study	Overall: 30 Male: 19	72.96 (50–86)	**Inclusion criteria:** patients aged 18–75 years requiring carotid endarterectomy for >70% stenosis **Exclusion criteria:** patients requiring combined aorto-coronary bypass surgery and carotid endarterectomy	Hypertension: 28 Hyperlipidaemia: 17 CAD: 12 Diabetes mellitus: 9 Chronic kidney disease: 3 Peripheral arterial disease: 2 Abdominal aorta aneurysm: 1
**Ebrahimian et al.** [26] Single-centre study	Overall: 85 Male: 56	73 ± 10	**Inclusion criteria:** patients undergoing dual-energy CTA of the neck to investigate common or internal carotid artery stenosis **Exclusion criteria:** patients scanned using other scanners, previous revascularisation surgery, metallic implants or stents, dental implants, motion artefact on imaging	DNM
**Kafouris et al.** [36] Single-centre study	Overall: 21 Male: 18	70.4 ± 7.0	**Inclusion criteria:** patients undergoing carotid endarterectomy for stenosis > 70% **Exclusion criteria:** cardiological ischaemic events < 6 months ago; active infection, inflammatory or neoplastic disease, uncontrolled diabetes mellitus, multiple significant stenoses across the carotid arteries	Hypertension: 18 Hyperlipidaemia: 15 Smoker: 11 Diabetes mellitus: 9 CAD: 4
**Liu et al.** [37] Multi-centre study	Overall: 280 Male: 201	**Symptomatic patients** Training group: 63.8 ± 7.2 Validation group: 63.0 ± 7.1 External test group: 62.8 ± 7.5 **Asymptomatic patients** Training group: 65.3 ± 8.8 Validation group: 61.0 ± 8.0 External test group: 63.4 ± 8.6	**Inclusion criteria:** extracranial carotid artery stenosis secondary to atherosclerosis disease **Exclusion criteria:** history of carotid stenting and endarterectomy, cardiac thrombus, carotid occlusion, poor image quality, symptomatic bilateral carotid stenosis	Hypertension: 209 Smoker: 202 CAD: 159 Hyperlipidaemia: 132 Diabetes mellitus: 99
**Nie et al.** [38] Single-centre study	Overall: 203 Male: 115	71.9 ± 9.6	**Inclusion criteria:** extracranial carotid atherosclerosis **Exclusion criteria:** ischemic stroke or TIA caused by intracranial carotid stenosis >50%, ischemic stroke or TIA occurred >2 weeks before CTA, posterior circulation symptoms, history of intervention to the cervicocerebral artery, cerebral haemorrhage, meningioma, craniotomy, arteriovenous fistula, temporal lobectomy, moyamoya disease, reversible cerebral vasoconstriction syndrome, arteritis, carotid artery dissection, carotid artery aneurysm, carotid artery web, poor image quality, incomplete clinical information	Hypertension: 155 Diabetes mellitus: 72 Smoker: 55 Hyperlipidaemia: 50
**Le et al.** [39] Single-centre study	Overall: 41 Male: 32	74.1 ± 8.4	**Inclusion criteria:** bilateral carotid atherosclerosis (Evans et al. [44]), nil inclusion criteria (Tarkin et al. [45]), DNM (Joshi et al. [46]) **Exclusion criteria:** atrial fibrillation (Evans et al. [44]), nil exclusion criteria (Tarkin et al. [45]), DNM (Joshi et al. [46])	Stroke: 30 Smoker: 29 (includes current and ex-smokers) Hypertension 27 TIA: 11 Diabetes mellitus: 8
**Shan et al.** [40] Single-centre study	Overall: 74 Male: 63	66.9 ± 8.8	**Inclusion criteria:** patients aged >18 years with carotid atherosclerotic plaque diagnosed on CTA and contrast-enhanced ultrasound **Exclusion criteria:** incomplete clinical information, poor image quality	Hypertension: 52 Smoker: 41 Diabetes mellitus: 29
**Shi et al.** [41] Single-centre study	Overall: 167 Male: 131	66.2 ± 7.7	**Inclusion criteria:** patients with suspected stroke who underwent head and neck CTA and brain MRI **Exclusion criteria:** incomplete clinical information, negative carotid CTA, cerebral haemorrhage, intra-cranial tumour, intra-cranial trauma, previous brain surgery, posterior circulation stroke, suspected cardioembolic	Hypertension: 115 Smoker: 91 Hyperlipidaemia: 73 Diabetes mellitus: 48 CAD: 23
**Xia et al.** [42] Single-centre study	Overall: 179 Male: 125	65.4 ± DNM	**Inclusion criteria:** patients undergoing carotid CTA with carotid artery stenosis of 30–50% **Exclusion criteria:** carotid artery dissection or aneurysm, intracranial vascular disease (e.g., intracranial atherosclerosis with stenosis < 50%, vasculitis, aneurysm), posterior circulation stroke, intracerebral haemorrhage; other causes of haemorrhagic stroke (e.g., cardioembolic source and chest embolism); patients with other neurological diseases such as brain tumours or demyelinating disease	DNM
**Coronary studies**				
**Chen et al.** [16] Single-centre study	Overall: 155 Male: 81	62 ± 10	**Inclusion criteria:** patients with suspected CAD who underwent plain CT and CTCA **Exclusion criteria:** patients without diabetes, previous history of coronary artery disease, history of cardiac or coronary surgery, anomalous origin of coronary artery, coronary malformation, coronary artery aneurysm, coronary artery calcium score >600, poor image quality	Hypertension: 113 Hyperlipidaemia: 54 Smoker: 31
**Chen et al.** [17] Multi-centre study	Overall: 214 Male: 163	Development group: 63 ± 11 Validation group: 65 ± 10	**Inclusion criteria:** minimum of 2 CTCA studies 6 months apart, baseline coronary artery stenosis was 25% to 70% **Exclusion criteria:** patients undergoing coronary artery bypass grafting or percutaneous coronary intervention before or during the study, missing or insufficient imaging data, poor image quality, different tube voltage settings used between the CTCA examinations	Hypertension: 147 Diabetes mellitus: 68 Hyperlipidaemia: 33 Smoker: 30
**Feng et al.** [18] Single-centre study	Overall: 280 Male: 184	Progression group: 70.1 ± 10.5 Non-progression group: 70.2 ± 10.0	**Inclusion criteria:** ≥2 CTCA examination ≥2 years apart with >2 mm atherosclerotic lesion on the baseline imaging, consistent imaging technique during both scans **Exclusion criteria:** incomplete clinical information, poor imaging quality, coronary revascularisation before or during the study	Hypertension: 223 Diabetes mellitus: 87 Smoker: 76
**Homayounieh et al.** [19] Single-centre study	Overall: 106 Male: 68	64 ± 7	**Inclusion criteria:** patients undergoing low-dose CT for lung cancer screening received CTCA within 12 months **Exclusion criteria:** coronary stents, prior cardiac surgery, metal artefacts in the cardiac region	Hyperlipidaemia: 91 Hypertension: 84 Smoker: 45 Diabetes mellitus: 28
**Hou et al.** [20] Single-centre study	Overall: 96 Male: 68	62.6 ± 13.4	**Inclusion criteria:** patients with suspected or known CAD who underwent CTCA and SPECT-myocardial perfusion imaging **Exclusion criteria:** poor image quality, no lesion on CTCA, previous ACS or revascularisation, MPI was conducted over 30 days after CTCA, failed automatic image segmentation	Hypertension: 61 Diabetes mellitus: 32 Smoker: 30 Hyperlipidaemia: 24
**Hu et al.** [21] Single-centre study	Overall: 109 Male: 81	**Training group** FFR ≤ 0.8 patients: 62.5 ± 8.3 FFR > 0.8 patients: 61.2 ± 8.2 **Validation group** FFR ≤ 0.8 patients: 71.3 ± 7.8 FFR > 0.8 patients: 66.6 ± 6.4	**Inclusion criteria:** patients who experienced non-emergency invasive coronary angiography and FFR within 30 days after CTCA examination, and target lesions were located in the epicardial coronary artery with a diameter > 2 mm **Exclusion criteria:** prior stent implantation, inadequate image quality, unsuccessful image segmentation, stenosis <30% or >90% in the target lesion, tandem lesions that precluded identification of the culprit lesion, previous cardiac resynchronisation or catheter ablation therapy, complex congenital heart disease, severe cardiac insufficiency or liver and kidney dysfunction, contraindication to iodine contrast and coronary microangiopathy	Hypertension: 81 Diabetes mellitus: 40 Hyperlipidaemia: 78 Smoker: 33
**Jing et al.** [22] Single-centre study	Overall: 620 Male: 336	**Training group** CAD patients: 53 (47–58) CCS patients: 63 (55–69) ACS patients: 59.7 ± 11.9 **Testing group** No CAD patients: 54 (49–58.3) CCS patients: 58 (53–69.8) ACS patients: 60.7 ± 10.9	**Inclusion criteria:** no history of ACS or coronary bypass surgery or stenting, absence of atrial fibrillation, no severe renal impairment (eGFR > 30ml/m/1.73 m^2^, no contraindication to iodine contrast; CTCA within 3 days followed by invasive coronary angiography **Exclusion criteria:** incomplete imaging and clinical data, coronary artery malformations, artificial valve, cardiac pacemaker, myocarditis, vasculitis, inadequate image quality	Hyperlipidaemia: 379 Hypertension: 362 Smoker: 286 Diabetes mellitus: 182
**Kim et al.** [23] Single-centre study	Overall: 25 Male: 19	63 ± 11	**Inclusion criteria:** patients that underwent both CTCA and IVOCT for the investigation of coronary plaques **Exclusion criteria:** history of myocardial infarction, previous coronary stent implantation, inadequate CTCA or IVOCT images	Hyperlipidaemia: 24 Diabetes mellitus: 20 Hypertension: 11 Chronic kidney disease: 11
**Kwiecinski et al.** [24] Multi-centre study	Overall: 260 Male: 216	65 ± 9	**Inclusion criteria:** patients with established CAD **Exclusion criteria:** coronary artery stenting	Hyperlipidaemia: 235 Smoker: 172 Hypertension: 153 Diabetes mellitus: 54 Peripheral arterial disease: 14
**Lee et al.** [25] Multi-centre study	Overall: 1162 Male: 647	60.3 ± 9.2	**Inclusion criteria:** patients that underwent clinically indicated CTCA **Exclusion criteria:** inadequate imaging quality, coronary revascularisation before or during the study, failure to extract radiomic features, coronary plaque at baseline	Hypertension: 600 Smoker: 431 Hyperlipidaemia: 420 Diabetes mellitus: 231
**Li et al.** [27] Single-centre study	Overall: 44 Male: 40	Training group: 53.0 ± 9.0 Validation group: 48.5 ± 11.6	**Inclusion criteria:** patients with CAD and end-stage heart failure who underwent CTCA prior to surgery **Exclusion criteria:** contraindications to CTCA, inadequate image quality	Hyperlipidaemia: 29 Smoker: 21 Hypertension: 17 Diabetes mellitus: 12
**Li et al.** [28] Multi-centre study	Overall: 132 Male: 91	Subtotal occlusion patients: 65 (55–71) Chronic total occlusion patients: 63 (58–73)	**Inclusion criteria:** patients with subtotal or chronic total coronary artery occlusion who underwent both CTCA and invasive coronary angiography **Exclusion criteria:** patients who underwent bypass surgery or percutaneous coronary intervention for occluded arteries, >2 week interval between CTCA and invasive coronary angiography, multiple occlusive lesions, excessive calcification precluding lumen analysis, inadequate image quality	Hypertension: 80 Diabetes mellitus: 48 Smoker: 48
**Lin et al.** [29] Single-centre study	Overall: 180 Male: 156	Acute MI patients: 58.4 (51.6–73.7) Stable CAD patients: 60.0 (52.0–68.5) No CAD patients: 59.5 (52.0–69.0)	**Inclusion criteria:** patients with post-thrombolysis STEMI or non-STEMI and had a culprit lesion identified on invasive coronary angiography **Exclusion criteria:** previous MI or revascularisation, clinical instability, severe renal impairment (eGFR < 30 ml/m/1.73 m^2^), allergy to iodinated contrast	Hypertension: 127 Diabetes mellitus: 40 Hyperlipidaemia: 98 Smoker: 63
**Lin et al.** [30] Single-centre study	Overall: 120 Male: 104	Acute MI patients: 59.9 ± 11.6 Stable CAD patients: 60.2 ± 11.3	**Inclusion criteria:** patients with acute MI undergoing CTCA and invasive coronary angiography **Exclusion criteria:** previous MI or revascularisation, clinical instability, severe renal impairment (eGFR < 30 ml/m/1.73 m^2^), allergy to iodinated contrast	Hypertension: 85 Hyperlipidaemia: 67 Smoker: 44 Diabetes mellitus: 28
**Oikonomou et al.** [31] Multi-centre study	Study 2 Overall: 202 Male: 134	MACE group: 64 (55–72) Non-MACE group: 62 (53–70)	**Inclusion criteria:** study 2—patients undergoing clinically indicated CTCA, study 3—patients undergoing CTCA after acute MI or stable CAD **Exclusion criteria:** DNM	Hypertension: 129 Hyperlipidaemia: 80 Smoker: 56 Diabetes mellitus: 34
	Study 3 Overall: 88 Male: 65	Stable CAD group: 62 (51–70) Acute MI group: 62 (53–72)		Smoker: 55 Hypertension: 42 Hyperlipidaemia: 41 Diabetes mellitus: 13
**Si et al.** [32] Single-centre study	Overall: 210 Male: 148	62.5 ± 10.4	**Inclusion criteria:** patients with acute MI **Exclusion criteria:** DNM	Hyperlipidaemia: 145 Hypertension: 111 Diabetes mellitus: 69 Smoker: 74
**Wen et al.** [33] Single-centre study	Overall: 92 Male: 66	58.3 ± 10.3	**Inclusion criteria:** patients suspected with CAD undergoing CTCA and invasive coronary angiography and FFR examination, <30-day interval between CTCA and FFR measurement **Exclusion criteria:** previous revascularisation, inadequate CTCA image quality, incomplete CTCA acquisition	Hypertension: 43 Hyperlipidaemia: 39 Smoker: 37 Diabetes mellitus: 8
**You et al.** [34] Multi-centre study	Overall: 288 Male: 175	**Training group** MACE patients: 59.1 ± 10.4 Non-MACE patients: 59.6 ± 9.6 **Validation group** MACE patients: 60.4 ± 10.0 Non-MACE patients: 61.4 ± 8.4	**Inclusion criteria:** patients who underwent CTCA—half of the cohort had a major adverse cardiovascular event within 3 years **Exclusion criteria:** previous PCI or CABG, revascularisation surgery within 6 weeks after CTCA, incomplete clinical information, inadequate imaging quality, previous MI, cardiomyopathy, valvular heart disease, congenital heart disease, chest malignancy	Hypertension: 193 Diabetes mellitus: 107 Smoker: 94 Hyperlipidaemia: 26
**Yu et al.** [35] Single-centre study	Overall: 146 Male: 102	65.5 ± 8.3	**Inclusion criteria:** patients with known CAD who had CTCA, invasive coronary angiography, and FFR within 1 month **Exclusion criteria:** previous revascularisation, tandem coronary lesions, previous MI, inadequate CTCA quality	Hypertension: 105 Hyperlipidaemia: 59 Diabetes mellitus: 56 Smoker: 50

Abbreviations: CTA = computed tomography angiogram, CAD = coronary artery disease, TIA = transient ischaemic attack, DNM = does not mention, MRI = magnetic resonance imaging, CT = computed tomography, CTCA = computed tomography coronary angiogram, SPECT = single-photon emission computed tomography, ACS = acute coronary syndrome, MPI = myocardial perfusion imaging, FFR = fractional flow reserve, CCS = chronic coronary syndrome, IVOCT = intra-vascular optical coherence tomography, MI = myocardial infarction, STEMI = ST elevation myocardial infarction, eGFR = estimated glomerular filtration rate, MACE = major adverse cardiovascular event, PCI = percutaneous coronary intervention and CABG = coronary artery bypass graft.

## Supplementary Materials

The following supporting information can be downloaded at: https://www.mdpi.com/article/10.3390/tomography10090108/s1, Table S1: PRISMA checklist [8]; Table S2: Quality assessment of the included studies using the Newcastle-Ottawa quality assessment tool [14,15,16,17,18,19,20,21,22,23,24,25,26,27,28,29,30,31,32,33,34,35,36,37,38,39,40,41,42]; Table S3: Imaging and radiomics methodology. Continuous variables displayed using mean ± standard deviation or median (interquartile range) [14,15,16,17,18,19,20,21,22,23,24,25,26,27,28,29,30,31,32,33,34,35,36,37,38,39,40,41,42]; Table S4: The radiomics analysis and comparative analysis in the studies [14,15,16,17,18,19,20,21,22,23,24,25,26,27,28,29,30,31,32,33,34,35,36,37,38,39,40,41,42].
