# Supplementary material for: A Scoping Review of Machine-Learning Derived Radiomic Analysis of CT and PET Imaging to Investigate Atherosclerotic Cardiovascular Disease
Source: Tomography. 2024 Sep 3;10(9):1455–87. doi: 10.3390/tomography10090108 (PMC11435603; doi:10.3390/tomography10090108)
Supplement: Supplementary file 1 [file tomography-10-00108-s001.zip › tomography-3150207-supplementary.pdf]

Supplementary Table S1: PRISMA checklist. Adapted from Tricco et al.[8]

Section	Item	PRISMA-Scoping Review Checklist Item	Reported on page #
**Title**			
**Title**	1	Identify the report as a scoping review.	Page 1
**Abstract**			
**Structured summary**	2	Provide a structured summary that includes (as applicable): background, objectives, eligibility criteria, sources of evidence, charting methods, results, and conclusions that relate to the review questions and objectives.	Page 2
**Introduction**			
**Rationale**	3	Describe the rationale for the review in the context of what is already known. Explain why the review questions/objectives lend themselves to a scoping review approach.	Page 3-5
**Objectives**	4	Provide an explicit statement of the questions and objectives being addressed with reference to their key elements (e.g., population or participants, concepts, and context) or other relevant key elements used to conceptualize the review questions and/or objectives.	Page 5
**Methods**			
**Protocol and registration**	5	Indicate whether a review protocol exists; state if and where it can be accessed (e.g., a Web address); and if available, provide registration information, including the registration number.	N/A
**Eligibility criteria**	6	Specify characteristics of the sources of evidence used as eligibility criteria (e.g., years considered, language, and publication status), and provide a rationale.	Page 6
**Information sources**	7	Describe all information sources in the search (e.g., databases with dates of coverage and contact with authors to identify additional sources), as well as the date the most recent search was executed.	Page 6
**Search**	8	Present the full electronic search strategy for at least 1 database, including any limits used, such that it could be repeated.	Suppleme ntary material
**Selection of sources of evidence**	9	State the process for selecting sources of evidence (i.e., screening and eligibility) included in the scoping review.	Page 6

**Data charting process**	10	Describe the methods of charting data from the included sources of evidence (e.g., calibrated forms or forms that have been tested by the team before their use, and whether data charting was done independently or in duplicate) and any processes for obtaining and confirming data from investigators.	Page 6-7
**Data items**	11	List and define all variables for which data were sought and any assumptions and simplifications made.	Page 6-7
**Critical appraisal of individual sources of evidence**	12	If done, provide a rationale for conducting a critical appraisal of included sources of evidence; describe the methods used and how this information was used in any data synthesis (if appropriate).	N/A
**Synthesis of results**	13	Describe the methods of handling and summarizing the data that were charted.	Page 7
**Results**			
**Selection of sources of evidence**	14	Give numbers of sources of evidence screened, assessed for eligibility, and included in the review, with reasons for exclusions at each stage, ideally using a flow diagram.	Page 7-8
**Characteristics of sources of evidence**	15	For each source of evidence, present characteristics for which data were charted and provide the citations.	Page 7-8
**Critical appraisal within sources of evidence**	16	If done, present data on critical appraisal of included sources of evidence (see item 12).	N/A
**Results of individual sources of evidence**	17	For each included source of evidence, present the relevant data that were charted that relate to the review questions and objectives.	Page 7-16
**Synthesis of results**	18	Summarize and/or present the charting results as they relate to the review questions and objectives.	Page 7-16
**Discussion**			
**Summary of evidence**	19	Summarize the main results (including an overview of concepts, themes, and types of evidence available), link to the review questions and objectives, and consider the relevance to key groups.	Page 16-19
**Limitations**	20	Discuss the limitations of the scoping review process.	Page 20-21
**Conclusions**	21	Provide a general interpretation of the results with respect to the review questions and objectives, as well as potential implications and/or next steps.	Page 22
**Funding**			
**Funding**	22	Describe sources of funding for the included sources of evidence, as well as sources of funding for the scoping review. Describe the role of the funders of the scoping review.	N/A

Search strategy

1 radiomic*.mp. [mp=ti, ab, hw, tn, ot, dm, mf, dv, kf, fx, dq, bt, nm, ox, px, rx, ui, sy, ux, mx] (26879)
2 peripheral arter*.mp. [mp=ti, ab, hw, tn, ot, dm, mf, dv, kf, fx, dq, bt, nm, ox, px, rx, ui, sy, ux, mx] (82305)
3 iliac.mp. [mp=ti, ab, hw, tn, ot, dm, mf, dv, kf, fx, dq, bt, nm, ox, px, rx, ui, sy, ux, mx] (123101)
4 femoral.mp. [mp=ti, ab, hw, tn, ot, dm, mf, dv, kf, fx, dq, bt, nm, ox, px, rx, ui, sy, ux, mx] (395248)
5 profunda.mp. [mp=ti, ab, hw, tn, ot, dm, mf, dv, kf, fx, dq, bt, nm, ox, px, rx, ui, sy, ux, mx] (5214)
6 popliteal.mp. [mp=ti, ab, hw, tn, ot, dm, mf, dv, kf, fx, dq, bt, nm, ox, px, rx, ui, sy, ux, mx] (44899)
7 coronar*.mp. [mp=ti, ab, hw, tn, ot, dm, mf, dv, kf, fx, dq, bt, nm, ox, px, rx, ui, sy, ux, mx] (1316043)
8 carotid.mp. [mp=ti, ab, hw, tn, ot, dm, mf, dv, kf, fx, dq, bt, nm, ox, px, rx, ui, sy, ux, mx] (347913)
9 2 or 3 or 4 or 5 or 6 or 7 or 8 (2147967)
10 Positron emission tomography.mp. [mp=ti, ab, hw, tn, ot, dm, mf, dv, kf, fx, dq, bt, nm, ox, px, rx, ui, sy, ux, mx] (373048)
11 PET.mp. [mp=ti, ab, hw, tn, ot, dm, mf, dv, kf, fx, dq, bt, nm, ox, px, rx, ui, sy, ux, mx] (379424)
12 Computed tomograph*.mp. [mp=ti, ab, hw, tn, ot, dm, mf, dv, kf, fx, dq, bt, nm, ox, px, rx, ui, sy, ux, mx] (1117069)
13 CT.mp. [mp=ti, ab, hw, tn, ot, dm, mf, dv, kf, fx, dq, bt, nm, ox, px, rx, ui, sy, ux, mx] (1845032)
14 Magnetic resonance.mp. [mp=ti, ab, hw, tn, ot, dm, mf, dv, kf, fx, dq, bt, nm, ox, px, rx, ui, sy, ux, mx] (2523101)
15 Magnetic resonance imaging.mp. [mp=ti, ab, hw, tn, ot, dm, mf, dv, kf, fx, dq, bt, nm, ox, px, rx, ui, sy, ux, mx] (1812058)
16 MRI.mp. [mp=ti, ab, hw, tn, ot, dm, mf, dv, kf, fx, dq, bt, nm, ox, px, rx, ui, sy, ux, mx] (926288)
17 PET-CT.mp. [mp=ti, ab, hw, tn, ot, dm, mf, dv, kf, fx, dq, bt, nm, ox, px, rx, ui, sy, ux, mx] (135475)
18 10 or 11 or 12 or 13 or 14 or 15 or 16 or 17 (4886004)
19 1 and 9 and 18 (496)
20 remove duplicates from 19 (342)

Supplementary Table S2: Quality assessment of the included studies using the Newcastle-Ottawa quality assessment tool.

Study	Selection	Comparability	Outcome	Total score
**Carotid studies**				
**Chen et al.**[14]	2/2*†	0/2	1/1‡§	3/5
**Cilla et al.**[15]	2/2*†	0/2	1/1‡§	3/5
**Ebrahimian et al.**[26]	2/2*†	0/2	1/1‡§	3/5
**Kafouris et al.**[36]	2/2*†	0/0¶	1/1‡§	3/3
**Liu et al.**[37]	2/2*†	0/2	1/1‡§	3/5
**Nie et al.**[38]	2/2*†	0/2	1/1‡§	3/5
**Le et al.**[39]	2/2*†	0/2	1/1‡§	3/5
**Shan et al.**[40]	2/2*†	0/2	1/1‡§	3/5
**Shi et al.**[41]	2/2*†	0/2	1/1‡§	3/5
**Xia et al.**[42]	2/2*†	0/2	1/1‡§	3/5
**Coronary studies**				
**Chen et al.**[16]	2/2*†	0/2	1/1‡§	3/5
**Chen et al.**[17]	2/2*†	2/2	1/1‡§	5/5
**Feng et al.**[18]	2/2*†	0/2	1/1‡§	3/5
**Homayounieh et al.**[19]	2/2*†	0/2	1/1‡§	3/5
**Hou et al.**[20]	2/2*†	0/2	1/1‡§	3/5
**Hu et al.**[21]	2/2*†	0/2	1/1‡§	3/5
**Jing et al.**[22]	2/2*†	0/2	1/1‡§	3/5
**Kim et al.**[23]	2/2*†	0/0¶	1/1‡§	3/3
**Kwiecinski et al.**[24]	3/3*	0/0¶	2/3	5/6
**Lee et al.**[25]	3/3*	1/1	2/3	5/7
**Li et al.**[27]	2/2*†	0/2	1/1‡§	3/5
**Li et al.**[28]#	4/4	2/2	2/2‖	8/8
**Lin et al.**[29]#	4/4	2/2	2/2‖	8/8
**Lin et al.**[30]#	4/4	2/2	2/2‖	8/8
**Oikonomou et al.**[31] **Study 2**#	4/4	2/2	2/2‖	8/8
**Oikonomou et al.**[31] **Study 3**#	4/4	2/2	2/2‖	8/8
**Si et al.**[32]#	4/4	2/2	2/2‖	8/8

**Wen et al.**[33]	2/2*†	0/2	1/1‡§	3/5
**You et al.**[34]#	4/4	2/2	2/2||	8/8
**Yu et al.**[35]	2/2*†	0/2	1/1‡§	3/5

Note: * A non-exposed cohort was not applicable to this study. † Outcome of interest had already occurred as this was a retrospective study. ‡ An assessment of the length of follow up for the outcome to occur was not performed as the study was retrospective in nature. § An assessment of loss to follow up was not performed as the study was retrospective in nature. ¶ Not applicable as a control cohort was not selected. # Case-control study. | | An assessment of the non-response rate was not applicable.

Supplementary Table S3: Imaging and radiomics methodology. Continuous variables displayed using mean ± standard deviation or median (interquartile range).

Study	Imaging technique	Radiomics architecture	Segmentation and processing	Performance evaluation
**Carotid studies**				
**Chen et al.**[14]	**CT angiography** using 60–80 ml of iodine contrast injected at 4-5 ml/s<br>**Scanner:** 64-row CT scanner (SOMATOM Definition AS+, Siemens)<br>**Images acquired** using embolic tracking 2 seconds after 100 HU reached in the aortic arch at the level of the tracheal bifurcation<br>**Tube voltage:** 100 kV<br>**Tube current:** 300 mA<br>**Matrix size:** 512 × 512<br>**Field of view:** 280 mm<br>**Slice thickness:** 0.6 mm<br>**Slice interval**: 0.5 mm	**Adherence to radiomics guidelines:** nil<br>**Feature extraction software:** 3D Slicer<br>**Hardware**: DNM	**Segmentation**: manual segmentation of the plaque and semiautomated segmentation of the PVAT using 3D Slicer by two radiologists. The plaque and PVAT (defined as adipose tissue in the radial distance from the outer wall of vessel equal to the vessel diameter ranging from -190 to -30HU) were drawn at the level of the maximum plaque area in the arterial phase on CTA. PVAT was drawn semiautomatically by setting an attenuation threshold<br>**Processing**: images resampled to 1×1×1 mm, discretised using a fixed bin width of 25 HU, and processed using Laplacian of Gaussian and Wavelet<br>**Features extracted:** shape, first order, GLCM, GLDM, GLSZM, GLRLM and NGTDM<br>**Feature selection**: features with ICC > 0.75 retained. t-test or Mann-Whitney U-test used to remove features with non-significant differences ($P < 0.05$). Then LASSO regression applied<br>**Machine learning techniques**: SVM	**Performance assessment:** AUC from the ROC, accuracy, sensitivity, specificity, PPV, and NPV<br>**Internal validation:** dataset split into training set (n = 100) and validation set (n = 44). Tenfold cross validation performed<br>**No external validation**
**Cilla et al.**[15]	**CT angiography** using 55 ml of iomeprol injected at 4 ml/s<br>**Scanner:** 128-slice CT scanner (Brilliance 128, Philips Healthcare)<br>**Images acquired** when attenuation of 140 HU reached in the ascending aorta	**Adherence to radiomics guidelines:** radiomic feature extraction performed in accordance with IBSI	**Segmentation**: manual segmentation of all CT slices by a radiologist and a vascular surgeon<br>**Processing**: nil<br>**Features extracted:** first order, shape, GLCM, GLRLM, GLSZM, NGTDM and GLDM	**Performance assessment:** AUC from the ROC, AUC, class-specific accuracy (proportion of both true positive and true

	**Tube voltage:** DNM<br>**Tube current:** DNM<br>**Matrix size:** DNM<br>**Field of view:** DNM<br>**Slice thickness:** 0.9 mm<br>**Slice interval**: DNM	**Feature extraction software:** Moddicom (radiomics software package for R)<br>**Hardware**: DNM	**Feature selection:** Spearman's rank correlation coefficient used to remove correlated features (ϱ ≥ 0.8). Then univariate analysis performed to select features associated with plaque classification (P <0.05). Finally, stepwise backward elimination applied to remove non-significant features<br>**Machine learning techniques:** logistic regression, SVM, CART	negatives amongst all cases), PPV, sensitivity and *F*-measure<br>**Internal validation:** fivefold cross validation applied to each machine learning model<br>**No external validation**
**Ebrahimian et al.**[26]	**Dual-energy CT angiography** using 80-100 ml of iohexol injected at 5 ml/s<br>**Scanner:** dual source 128-slice CT scanner (Siemens Definition Flash, Siemens Healthineers)<br>**Images acquired** when the contrast bolus reaches the ascending aorta<br>**Tube voltage:** dual-energy scan mode using 80 kV (tube A) and 140 kV with tin filter (tube B)<br>**Tube current:** 320 mA (tube A), the system automatically selects the corresponding value for tube B<br>**Matrix size:** DNM<br>**Field of view:** DNM<br>**Slice thickness:** 1 mm **Slice interval**: 0.5 mm	**Adherence to radiomics guidelines:** nil<br>**Feature extraction software:** PyRadiomics integrated into Dual-Energy Tumour Analysis prototype software (eXamine, Siemens Healthineers)<br>**Hardware**: DNM	**Segmentation**: automated segmentation using Dual-Energy Tumour Analysis prototype software (eXamine, Siemens Healthineers). Segmentation software integrates all three imaging planes to extract spectral and radiomic features over both the section with maximal stenosis and the entire length of stenosis. No assessment of the accuracy of the segmentation software<br>**Processing**: images discretised using a fixed bin width of 25 HU<br>**Features extracted:** shape, first-order, GLCM, NGTDM, GLSZM, GLRLM, GLDM, and higher-order features<br>**Feature selection:** t-test and ANOVA used to identify statistically significant features (P < 0.05). Then MRMR algorithm applied to select the most relevant features. Finally, stepwise forward selection applied to identify the best feature subsets<br>**Machine learning techniques:** multinomial logistic regression	**Performance assessment:** AUC from the ROC<br>**Internal validation:** DNM<br>**No external validation**
**Kafouris et al.**[36]	**PET/CT** using 0.14 mCi/kg ^{18}F-FDG<br>**Scanner:** Biograph 6 (Siemens)<br>**Image acquisition method:** DNM<br>**Tube voltage:** 110 kV	**Adherence to radiomics guidelines:** features extracted	**Segmentation**: manual segmentation. ROIs placed around the carotid artery wall on each axial CT slice guided by the co-registered PET/CT images<br>**Processing**: image SUVs were discretised into 64 bins	**Performance assessment:** AUC from the ROC

	**Tube current:** 30 mA<br>**Matrix size:** DNM<br>**Field of view:** DNM<br>**Slice thickness:** 1.25 mm<br>**Slice interval:** DNM	according to IBSI guidelines<br>**Feature extraction software:** in-house software based on Matlab platform (Version 9.3, Matlab R2017b)<br>**Hardware**: DNM	**Features extracted:** first order, GLCM, GLRLM, GLSZM and NGTDM<br>**Feature selection:** Univariate logistic regression analysis used to identify features that can significantly predict the outcome (P < 0.157). Then Spearman's rank correlation coefficient used to remove correlated features (ϱ ≥ 0.8)<br>**Machine learning techniques:** univariate logistic regression	**Internal validation:** bootstrapping generating 200 bootstrap samples<br>**No external validation**
**Liu et al.**[37]	**CT angiography** using 70-90 ml of iohexol injected at 5-6 ml/s<br>**Scanner:** dual-source CT scanner (SOMATOM Force; Siemens Healthineers)<br>**Images acquired** when attenuation of 100 HU reached in the aortic arch<br>**Tube voltage:** 110 kV<br>**Tube current:** DNM<br>**Matrix size:** DNM<br>**Field of view:** DNM<br>**Slice thickness:** 0.5 mm<br>**Slice interval**: 0.5 mm	**Adherence to radiomics guidelines:** nil<br>**Feature extraction software:** Radcloud platform (Huiying Medical Technology)<br>**Hardware**: DNM	**Segmentation**: manual segmentation using ITK-SNAP software (version 3.7) by two radiologists. ROIs placed within the border of the plaque. The inter- and intra-class correlation tested by repeated segmentation of the same images at a later date<br>**Processing**: nil<br>**Features extracted:** shape, first order, GLDM, GLRLM, GLCM, GLSZM and NGTDM<br>**Feature selection:** features with ICC > 0.75 retained. Then ANOVA used to identify significant features. Then LASSO applied to select the best features<br>**Machine learning techniques:** LASSO used to construct a 'radiomics score'	**Performance assessment:** AUC from the ROC<br>**Internal validation:** dataset split into training set (n = 135) and validation set (n = 58)<br>**External validation using 87 patients**
**Nie et al.**[38]	**CT angiography** using 50 ml of ioversol injected at 5 ml/s<br>**Scanner:** dual-source CT scanner (SOMATOM Force; Siemens Healthineers)<br>**Images acquired** when attenuation of 100 HU reached in the descending aortic arch<br>**Tube voltage:** 90-100 kV<br>**Tube current:** adaptive<br>**Matrix size:** DNM	**Adherence to radiomics guidelines:** nil<br>**Feature extraction software:** Shukun AI Scientific Research Platform (Shukun Technology)<br>**Hardware**: DNM	**Segmentation**: automated segmentation using perivascular fat analysis software (Shukun Technology). The PVAT (defined as the equivalent diameter of the carotid artery beyond the outer wall of the vessel) with attenuation of -190 HU to -30 HU was segmented along the target length and width of the vessel. No assessment of the accuracy of the segmentation software<br>**Processing**: nil<br>**Features extracted:** first order, shape, GLCM, GLDM, GLRLM, GLSZM and NGTDM	**Performance assessment:** AUC from the ROC<br>**Internal validation:** dataset split into training set (n = 163) and test set (n = 40)<br>**No external validation**

	**Field of view:** DNM<br>**Slice thickness:** DNM<br>**Slice interval:** DNM		**Feature selection:** MRMR algorithm and recursive feature algorithm applied to select the most relevant features<br>**Machine learning techniques:** Bagging DecisionTree, XGBoost, random forest, SVM and quadratic discriminant analysis	
**Le et al.**[39]	**CT angiography** using 70-100 ml iopamidol injected at 5 ml/s<br>**Scanner:** PET/CT combined scanner with an integrated 64-slice CT scanner (GE Discovery combined 690, GE Healthcare)<br>**Images acquired** when attenuation of 100 HU reached in the aortic arch<br>**Tube voltage:** 120 kV<br>**Tube current:** 200 mA<br>**Matrix size:** DNM<br>**Field of view:** DNM<br>**Slice thickness:** 0.625 mm<br>**Slice interval:** 0.4 mm	**Adherence to radiomics guidelines: nil**<br>**Feature extraction software:** PyRadiomics (version 3.0)<br>**Hardware**: DNM	**Segmentation**: manual segmentation using TexRad (Feedback Medical Ltd) performed on single slice and multi-slice axial images. The ROI was eroded and dilated (single slice = circular structuring element of radius 1 with iterations of 1–2 for ROI dilation and erosion, multi slice = spherical structuring element of radius 1, with iterations of 1–2 for ROI dilation and 1 iteration for ROI erosion)<br>**Processing**: normalisation of images so that the pixel values assumed an approximate Gaussian distribution, resegmentation applied with a lower limit of 0 HU and upper limit of 200 HU<br>**Features extracted:** first order, GLCM, GLRLM, GLSZM, GLDM, and NGTDM<br>**Feature selection:** features with ICC ≥ 0.9 retained. Then Spearman's rank correlation used to assess feature collinearity. For feature pairs with $r_s \geq 0.95$, the feature with the highest AUC in univariate logistic regression was retained. Finally, features were standardised to have a mean of zero and a variance of one<br>**Machine learning techniques:** decision tree, random forest, LASSO, Elastic Net regression (weight for $L1$ and $L2$ penalties = 0.5), neural network, and XGBoost	**Performance assessment:** AUC from the ROC<br>**Internal validation:** fivefold cross validation performed<br>**No external validation**
**Shan et al.**[40]	**CT angiography**<br>**Scanner:** 320 × 0.5-mm detector row CT scanner (AquilionONE, Canon Medical Systems)	**Adherence to radiomics guidelines:** nil	**Segmentation**: semi-automated segmentation using 3D Slicer by two radiologists. No assessment of the accuracy of the segmentation software	**Performance assessment:** AUC from the ROC

	**Image acquisition method: DNM**<br>**Tube voltage:** 80 kV<br>**Tube current:** 100 mA<br>**Matrix size:** DNM<br>**Field of view:** 16 cm<br>**Slice thickness:** 0.5 mm	**Feature extraction software:** PyRadiomics integrated into Python<br>**Hardware**: DNM	**Processing**: images processed using Laplacian of Gaussian, wavelet decomposition, and exponential and gradient filters to reveal further features<br>**Features extracted:** shape, first order, GLDM, GLRLM, GLCM, GLSZM and NGTDM<br>**Feature selection:** features with ICC > 0.9 retained. Then t-test used to identify statistically significant different features between vulnerable and stable patients (P < 0.05)<br>**Machine learning techniques:** logistic regression, SVM, random forest, light gradient boosting machine, AdaBoost, XGBoost, and multi-layer perception	**Internal validation:** dataset split into training set and validation set in a ratio of 7:3<br>**No external validation**
**Shi et al.**[41]	**CT angiography** using 50-60 ml of non-ionic iodine contrast injected at 4-5 ml/s<br>**Scanner:** 256-slice CT scanner (Brilliance iCT; Philips Medical Systems)<br>**Image acquisition method:** DNM<br>**Tube voltage:** 120 kV<br>**Tube current:** 250 mA<br>**Matrix size:** DNM<br>**Field of view:** DNM<br>**Slice thickness:** 0.9 mm<br>**Slice interval:** 0.45 mm	**Adherence to radiomics guidelines:** nil<br>**Feature extraction software:** The Deepwise Multimodal Research Platform (version 2.0, Beijing Deepwise & League of PHD Technology Co. Ltd)<br>**Hardware**: DNM	**Segmentation**: manual segmentation using The Deepwise Multimodal Research Platform by one radiologist. The outer border of the arterial wall was visually assessed and the plaque was accordingly segmented<br>**Processing**: nil<br>**Features extracted:** shape, first order, GLDM, GLRLM, GLCM, GLSZM and NGTDM<br>**Feature selection:** features with ICC > 0.8 retained. Then features with Pearson's correlation coefficient <0.7 removed<br>**Machine learning techniques:** analysis of variance F-value, mutual information and linear models penalised with the L1 norm	**Performance assessment:** AUC from the ROC, calibration, and decision curve analyses<br>**Internal validation:** fivefold cross validation applied to each machine learning model<br>**No external validation**
**Xia et al.**[42]	**CT angiography** using 60-80 ml of iopromide injected at 5 ml/s<br>**Scanner:** SOMATOM Definition Flash dual-source CT scanner<br>**Images acquired** when attenuation of 150 HU reached in the aortic arch<br>**Tube voltage:** 120 kV	**Adherence to radiomics guidelines:** nil<br>**Feature extraction software:** PyRadiomics (version	**Segmentation**: manual segmentation using 3D Slicer (version 4.11) by one radiologist. The carotid artery with plaque was manually segmented into the targeted region<br>**Processing**: nil<br>**Features extracted:** shape, first order, GLCM, GLSZM, GLRLM, NGTDM and GLDM	**Performance assessment:** predictive value of the model assessed using AUC from the ROC<br>**Internal validation:** dataset split into

	**Tube current:** DNM<br>**Matrix size:** DNM<br>**Field of view:** DNM<br>**Slice thickness:** 0.625 mm<br>**Slice interval:** 0.625 mm	2.4) integrated into Python<br>**Hardware**: DNM	**Feature selected:** random forest algorithm used to select features<br>**Machine learning techniques:** random forest, XGBoost, logistic regression, SVM and k-nearest neighbour	training set (n = 165) and validation set (n = 66). Fivefold cross validation used on the training set<br>**No external validation**
**Coronary studies**				
**Chen et al.**[16]	**CT coronary angiography** using 45 ml of iopromide injected at 5 ml/s<br>**Scanner:** 256-row CT scanner (Revolution CT, GE Healthcare)<br>**Images acquired** when attenuation of 220 HU reached in the ascending aorta<br>**Tube voltage:** 100 kV<br>**Tube current:** 599 mA<br>**Matrix size:** DNM<br>**Field of view:** DNM<br>**Slice thickness:** 0.625 mm<br>**Slice interval:** 0.625 mm	**Adherence to radiomics guidelines:** nil<br>**Feature extraction software:** Perivascular Fat Analysis Tool (Shukun Technology)<br>**Hardware**: DNM	**Segmentation**: semi-automated segmentation using Perivascular Fat Analysis Tool by two radiologists. The PCAT was defined as all voxels ranging from −190 to −30 HU located within a radial distance from the outer vessel wall equal to the diameter of the vessel. No assessment of the accuracy of the segmentation software<br>**Processing**: wavelet and Laplacian of Gaussian filters, and non-linear strength transformation<br>**Features extracted**: shape, first order, GLDM, GLCM, GLRLM, GLSZM and NGTDM<br>**Feature selection:** features with ICCs ≥ 0.9 retained. LASSO regression performed to reduce the number of features<br>**Machine learning techniques:** multivariate logistic regression used to construct a 'radiomics score'	**Performance assessment:** AUC from the ROC<br>**Internal validation:** dataset split into training set (n = 108) and validation set (n = 47). Fivefold cross validation performed<br>**No external validation**
**Chen et al.**[17]	**CT coronary angiography** using 60-70 ml of iopromide injected at 4-5 ml/s<br>**Scanner:** two dual-source CT scanners (Somatom Definition and Definition Flash, Siemens)<br>**Image acquisition method:** DNM<br>**Tube voltage:** 100-120 kV<br>**Tube current:** DNM<br>**Matrix size:** 512×512<br>**Field of view**	**Adherence to radiomics guidelines:** features extracted according to IBSI guidelines<br>**Feature extraction software:** Radiomics, Syngo.Via FRONTIER (version 1.2.1, Siemens)	**Segmentation**: manual segmentation using Radiomics, Syngo.Via FRONTIER<br>**Processing**: images resampled to 1×1×1 mm, B-spline interpolation applied and discretisation of images to bin width 25 HU<br>**Features extracted**: shape, first order, GLCM, GLSZM, GLRLM, GLDM and NGTDM<br>**Feature selection:** features with ICC ≥0.8 retained. Then agglomerative clustering with Ward's linkage and	**Performance assessment:** predictive value of the model assessed using AUC from the ROC<br>**Internal validation:** dataset split into training set and validation set in a ratio of 7:3. Fivefold

	**Slice thickness:** 0.75 mm<br>**Slice interval:** 0.5 mm	**Hardware**: DNM	Spearman's rank correlation used to retain the best features in each cluster (lowest P value in each cluster)<br>**Machine learning techniques:** multivariable logistic regression and XGBoost used to construct the algorithm	cross validation used on the training set (n = 137)<br>**External validation using 159 patients**
**Feng et al.**[18]	**CT coronary angiography** using 50-80 ml of iopromide injected at 4-6 ml/s<br>**Scanner:** dual-source CT scanner (Somatom Definition, Siemens Medical Systems)<br>**Image acquisition method:** DNM<br>**Tube voltage:** 120 kV<br>**Tube current:** DNM<br>**Matrix size:** DNM<br>**Field of view:** DNM<br>**Slice thickness:** 0.75 mm<br>**Slice interval:** 0.5 mm	**Adherence to radiomics guidelines:** nil<br>**Feature extraction software:** Radiomics, Syngo.Via FRONTIER (version 1.3.0, Siemens)<br>**Hardware**: DNM	**Segmentation**: semi-automated segmentation using Coronary Plaque Analysis Syngo.Via Frontier (version 5.0.2, Siemens Healthineers) by two radiologists. The proximal and distal ends of the plaque and area of maximum stenosis were manually defined whilst the software semiautomatically outlined the inner and outer contours of the plaque and the blood vessels. No assessment of the accuracy of the segmentation software<br>**Processing**: wavelet and Laplacian of Gaussian filtering, and non-linear intensity transformation applied<br>**Features extracted**: shape, first order and texture<br>**Feature selection:** Boruta algorithm used to identify the 10 most important features<br>**Machine learning techniques:** random forest model and logistic regression used to construct the radiomics mode	**Performance assessment:** AUC from the ROC, sensitivity, specificity, and accuracy<br>**Internal validation:** dataset split into training set (n = 280) and validation set (n =120)<br>**No external validation**
**Homayounieh et al.**[19]	**CT coronary angiography**<br>**Scanner:** 256-detector-row single source CT scanner (GE Revolution, GE Healthcare), n = 55; 64-detector-row single-source CT scanner (Philips IQon, Philips Healthcare), n = 36;, and 96-detector-row dual-source CT scanner (Siemens Definition Force, Siemens Healthineers), n = 15<br>**Image acquisition method:** DNM<br>**Tube voltage:** 120 kV<br>**Tube current:** 30-50 mA	**Adherence to radiomics guidelines:** nil<br>**Feature extraction software:** Radiomics, Syngo.Via FRONTIER (Siemens Healthineers)<br>**Hardware**: DNM	**Segmentation**: automated segmentation using Radiomics, Syngo.Via FRONTIER. No assessment of the accuracy of the segmentation software<br>**Processing**: nil<br>**Features extracted:** shape, first order, GLCM, GLRLM, GLSZM, NGTDM and GLDM<br>**Feature selection:** radiomics extraction software automatically selected the best radiomic features<br>**Machine learning techniques:** multiple logistic regression and kernel Fisher discriminant analysis	**Performance assessment:** AUC from the ROC<br>**Internal validation:** nil<br>**No external validation**

	**Matrix size:** DNM<br>**Field of view:** DNM			
**Hou et al.**[20]	**CT coronary angiography**<br>**Scanner:** 64 detector row CT scanner (Somatom Flash, Siemens Healthineers)<br>**Images acquired** when attenuation of 200 HU reached in the aortic arch<br>**Tube voltage:** 100 kV<br>**Tube current:** 350-500 mA<br>**Matrix size:** DNM<br>**Field of view:** DNM<br>**Slice thickness:** 0.5 mm<br>**Slice interval:** 0.25 mm	**Adherence to radiomics guidelines:** nil<br>**Feature extraction software:** DNM<br>**Hardware**: DNM	**Segmentation**: semi-automated segmentation of the pericoronary adipose tissue. Automated software identified the pericoronary adipose tissue, however this was manually adjusted by two radiologists. If multiple lesions were present on a single vessel, the adipose tissue around the highest-stenosis lesion was segmented. No assessment of the accuracy of the automated segmentation software<br>**Processing**: resampling of images to 1×1×1 mm^3, standardising of the grey level to 1-32 scales<br>**Features extracted:** first order, GLCM, GLRLM, GLSZM, GLDM and NGTDM<br>**Feature selection:** features with ICC > 0.75 retained. Then Mann-Whitney U-test and LASSO regression used to select the best features<br>**Machine learning techniques:** SVM, k-nearest neighbour, Light GBM, and random forest	**Performance assessment:** AUC from the ROC<br>**Internal validation:** dataset split into training set (n = 123) and validation set (n = 54). Tenfold cross validation used on the training set<br>**No external validation**
**Hu et al.**[21]	**CT coronary angiography**<br>**Scanner:** 64-slice dual-source CT scanne**r** (Siemens Somatom Flash, Siemens Sector Healthcare) used for the training set, 64-slice high-definition CT scanner (GE Discovery HD750, General Electric) used for the validation set<br>**Image acquisition method:** DNM<br>**Tube voltage:** DNM<br>**Tube current:** DNM<br>**Matrix size:** DNM<br>**Field of view:** DNM	**Adherence to radiomics guidelines:** nil<br>**Feature extraction software:** PyRadiomics library integrated into an unknown software<br>**Hardware**: DNM	**Segmentation**: manual segmentation using ITK-SNAP software (version 3.6.0) by two radiologists. Images sliced from the proximal to the distal end of the target lesion in perpendicular direction to the long axis of the target lesion. Then the lumen and the exterior borders of the blood vessel wall were manually delineated<br>**Processing**: nil<br>**Features extracted:** first order, shape, texture, higher order<br>**Feature selection:** features with ICC > 0.75 retained. Then LASSO regression used to select the best features<br>**Machine learning techniques:** logistic regression	**Performance assessment:** AUC from the ROC, sensitivity, specificity, PPV, NPV, positive likelihood ratio, negative likelihood ratio<br>**Internal validation:** dataset split into training set (n = 88) and validation set (n = 31)

				**No external validation**
**Jing et al.**[22]	**CT coronary angiography** using 0.9 ml/kg iopromide injected at 0.9 ml/kg<br>**Scanner:** Discovery CT 750HD (GE Healthcare)<br>**Image acquisition method:** DNM<br>**Tube voltage:** 120 kV<br>**Tube current:** 300-450mA<br>**Matrix size:** DNM<br>**Field of view:** DNM<br>**Slice thickness:** DNM<br>**Slice interval:** DNM	**Adherence to radiomics guidelines:** nil<br>**Feature extraction software:** PyRadiomics library integrated into Pericoronary Adipose Tissue Analysis software<br>**Hardware**: DNM	**Segmentation**: automated segmentation using CoronaryDoc software (China Shukun Technology Co.) based on multiple models.<br>The ResU-Net model focuses on coronary artery segmentation. The segmented coronary vessels are straightened and reconstructed at 1 mm intervals along the centrelines. Then, the second ResU-Net model is applied to segment the coronary artery lumens. The model was trained on ≥ 10,000 cases of patients ranging from 20-90 years and was verified on another independent dataset comprised of > 2000 cases<br>**Processing:** nil<br>**Features extracted:** first order and texture features<br>**Feature selection:** features with Spearman's rank correlation coefficient $\varrho < 0.3$ were removed. Then LASSO regression performed to select the best features<br>**Machine learning techniques:** SVM, ridge regression classifier and logistic regression	**Performance assessment:** AUC from the ROC, accuracy, specificity, sensitivity, PPV, and NPVs<br>**Internal validation:** dataset split into training set and validation set at a ratio of 2:1. Fivefold cross validation performed<br>**No external validation**
**Kim et al.**[23]	**CT coronary angiography** using 80 ml of iopamidol injected at 6 ml/s<br>**Scanner:** Brilliance ICT 256 scanner (Philips Healthcare)<br>**Images acquired** when attenuation of 100 HU reached in the ascending aorta at the level of the carina<br>**Tube voltage:** 100-120 kV<br>**Tube current:** 300-800 mA<br>**Matrix size:** DNM<br>**Field of view:** DNM<br>**Slice thickness:** DNM<br>**Slice interval:** DNM	**Adherence to radiomics guidelines:** features extracted according to IBSI guidelines<br>**Feature extraction software:** PyRadiomics integrated into Python<br>**Hardware**: DNM	**Segmentation**: semi-automated segmentation. First manual segmentation of the vessel walls using Aquarias. Then the PCAT from these delineated vessels was automatically identified (-190 to -30 HU) and segmented using in-house Python software. No assessment of the accuracy of the automated segmentation software<br>**Processing**: images were discretised to 8, 16, and 32 equally sized bins with identical HU ranges<br>**Features extracted:** shape, first order, GLCM, GLDM, GLRLM, GLSZM and NGTDM<br>**Feature selection:** radiomic features demonstrating a strong Pearon's correlation coefficient (r > 0.95) with	**Performance assessment:** predictive value of the model assessed using AUC from the ROC<br>**Internal validation:** stratified threefold cross validation performed<br>**No external validation**

			IVOCT features (thin-cap fibroatheroma and microchannels) were removed. Then each radiomic feature was assessed using univariate logistic regression and threefold cross validation to generate ROCs. The top 15 AUCs in each radiomic feature class (shape, first order, five texture features) were selected. MRMR used to reduce the number of features<br>**Machine learning techniques:** multivariate logistic regression	
**Kwiecinski et al.**[24]	**PET/CT** performed using 250 MBq ^{18}F-NaF<br>**Scanner:** 128-slice Biograph mCT (Siemens Medical Systems) or Discovery 710 (GE Healthcare)<br>**Image acquisition method:** DNM<br>**Tube voltage:** DNM<br>**Tube current:** DNM<br>**Matrix size:** DNM<br>**Field of view:** DNM<br>**Slice thickness:** DNM<br>**Slice interval:** DNM	**Adherence to radiomics guidelines:** nil<br>**Feature extraction software:** Radiomics Image Analysis (version 1.4.2) on R<br>**Hardware**: DNM	**Segmentation**: automated segmentation of the PET/CT using coronary microcalcification activity. Semi-automated segmentation of the CTCA using Autoplaque (version 2.5, Cedars-Sinai Medical Center). Proximal and distal ends of the lesions were manually marked. Subsequent plaque quantification was fully automated using adaptive scan-specific thresholds. No assessment of the accuracy of the automated segmentation software<br>**Processing**: nil<br>**Features extracted:** DNM type of features extracted<br>**Feature selection:** linear mixed models used to correct for intrapatient clustering and calculate the interpair similarity. Then, a hierarchical clustering dendogram was calculated and a dynamic tree-cut algorithm was used to identify the optimal number of feature clusters. Finally, the first principal component of each cluster was calculated<br>**Machine learning techniques:** univariable and multivariable logistic regression, linear regression and random forest	**Performance assessment:** nil<br>**Internal validation:** DNM<br>**No external validation**
**Lee et al.**[25]	**CT coronary angiography**<br>**Scanner:** DNM<br>**Image acquisition method:** DNM	**Adherence to radiomics guidelines:** nil	**Segmentation**: semi-automated segmentation of the coronary plaques using QAngioCT Research Edition (version 2.1.9.1, Medis Medical Imaging). No	**Performance assessment:** AUC from the ROC

	**Tube voltage:** DNM<br>**Tube current:** DNM<br>**Matrix size:** DNM<br>**Field of view:** DNM<br>**Slice thickness:** DNM<br>**Slice interval:** DNM	**Feature extraction software:** PyRadiomics integrated into Python<br>**Hardware**: DNM	assessment of the accuracy of the automated segmentation software<br>**Processing**: Nil<br>**Features extracted:** first order, GLCM, GLRLM, GLSZM, GLDM and NGTDM<br>**Feature selection:** features with concordance correlation coefficient < 0.85 were excluded. Then the Boruta and XGBoost algorithms used to select the final features<br>**Machine learning techniques:** multivariable Cox regression model	**Internal validation:** dataset split into training set and validation set in a ratio of 8:2<br>**No external validation**
**Li et al.**[27]	**CT coronary angiography** using 50-60 ml iopromide<br>**Scanner:** dual-source CT scanner (SOMATOM Definition Flash, Siemens Healthcare)<br>**Images acquired** when attenuation of 100 HU reached in the ascending aorta<br>**Tube voltage:** 80-120 kV<br>**Tube current:** 320-400 mA<br>**Matrix size:** DNM<br>**Field of view:** DNM<br>**Slice thickness:** DNM<br>**Slice interval:** DNM	**Adherence to radiomics guidelines:** nil<br>**Feature extraction software:** PyRadiomics integrated into Python<br>**Hardware**: DNM	**Segmentation**: manual segmentation of the coronary plaques on short-axis section by one radiologist<br>**Processing**: filtered using Laplacians of Gaussians, wavelet decompositions, square, square root, logarithm and exponential filters<br>**Features extracted:** shape, first order, GLCM, GLDM, GLRLM, GLSZM and NGTDM<br>**Feature selection:** LASSO regression used to select the best features<br>**Machine learning techniques:** Nai¨ve Bayes, decision tree, random forest, gradient boosting decision tree, SVM, multilayer perceptron, logistic regression, and k-nearest neighbours	**Performance assessment:** AUC from the ROC<br>**Internal validation:** dataset split into training set (n = 36) and validation set (n = 8). Fivefold cross validation performed on the training set<br>**No external validation**
**Li et al.**[28]	**CT coronary angiography** using 30-58 ml contrast injected at 3.8-5.8 ml/s<br>**Scanner:** second-generation dual-source CT scanner (SOMATOM Definition Flash, Siemens Healthineers), third-generation dual-source CT scanner (SOMATOM Force, Siemens Healthineers) or a 256-row wide-detector CT scanner (Revolution HD, GE Healthcare)	**Adherence to radiomics guidelines:** nil<br>**Feature extraction software:** PyRadiomics integrated into Research Portal (version 1.1, United	**Segmentation**: automated segmentation of the coronary plaques using Research Portal (version 1.1, United Imaging Intelligence Co. Ltd.). The initial segmentation was performed using the "RB-Net" network. For finer segmentation of the coronary tree, key topological information was constructed by combining a convolutional graph network with a point cloud network technique. Finally, a bidirectional recurrent convolutional neural network was used to detect the ROI. The ROI was reviewed by two radiologists.	**Performance assessment:** AUC from the ROC<br>**Internal validation:** dataset split into training set and validation set in a ratio of 8:2. Fivefold cross validation performed

	**Image acquisition method:** DNM<br>**Tube voltage:** DNM<br>**Tube current:** DNM<br>**Matrix size:** DNM<br>**Field of view:** DNM<br>**Slice thickness:** 0.75 mm<br>**Slice interval:** 0.5 mm	Imaging Intelligence Co. Ltd.)<br>**Hardware**: DNM	**Features extracted:** shape, first order, GLCM, GLRLM, GLSZM, NGTDM and GLDM<br>**Processing**: wavelet and Laplacian Sharpening filters applied<br>**Feature selection:** ANOVA used to identify significantly different radiomics features (P <0.05). Then LASSO regression used to select the best features<br>**Machine learning techniques:** DNM	**External validation using 50 patients**
**Lin et al.**[29]	**CT coronary angiography** using 60-90 ml iodine contrast injected at 5 ml/s<br>**Scanner:** DNM<br>**Image acquisition method:** DNM<br>**Tube voltage:** 100-120 kV<br>**Tube current:** 300-500 mA<br>**Matrix size:** DNM<br>**Field of view:** DNM<br>**Slice thickness:** 0.5 mm<br>**Slice interval:** 0.25 mm	**Adherence to radiomics guidelines:** nil<br>**Feature extraction software:** Radiomics Image Analysis software package (version 1.4.1) on R<br>**Hardware**: DNM	**Segmentation**: automated segmentation using Autoplaque software (version 2.5, Cedars-Sinai Medical Center). The PCAT was defined as all voxels ranging from -190 to -30 HU located within a radial distance from the outer coronary wall equal to the diameter of the vessel. No assessment of the accuracy of the automated segmentation software<br>**Processing**: images were discretised to 8, 16, and 32 equally sized bins with identical HU ranges<br>**Features extracted:** shape, first order features, GLCM and GLRLM<br>**Feature selection:** principal component analysis used to identify relevant features<br>**Machine learning techniques:** XGBoost	**Performance assessment:** AUC from the ROC<br>**Internal validation:** tenfold cross validation<br>**No external validation**
**Lin et al.**[30]	**CT coronary angiography** using 60-90 ml of iohexol injected at 5 ml/s<br>**Scanner:** 320-detector-row CT scanner (Aquilion ONE ViSION, Canon Medical Systems)<br>**Image acquisition method:** DNM<br>**Tube voltage:** 100-120 kV<br>**Tube current:** 300-500 mA<br>**Matrix size:** DNM<br>**Field of view:** DNM<br>**Slice thickness:** 0.5 mm	**Adherence to radiomics guidelines:** nil<br>**Feature extraction software:** Radiomics Image Analysis software package (version 1.4.2) on R<br>**Hardware**: DNM	**Segmentation**: semi-automated segmentation using Autoplaque (version 2.5, Cedars-Sinai Medical Center). No assessment of the accuracy of the automated segmentation software<br>**Processing**: images were discretised to 8, 16, and 32 equally sized bins with identical HU ranges<br>**Features extracted:** shape, first order, GLCM and GLRLM<br>**Feature selection:** principal component analysis used to identify relevant features<br>**Machine learning techniques:** XGBoost	**Performance assessment:** AUC from the ROC<br>**Internal validation:** tenfold cross validation<br>**External validation on 19 patients**

	**Slice interval:** 0.25 mm			
**Oikonomou et al.**[31]	This study constituted of multiple patient cohorts hence the salient datasets have been described separately			
**Study 2**	**CT coronary angiography performed** using iopamidol injected at 5-6 ml/s<br>**Scanner:** 256-slice Brilliance iCT scanner (Philips Medical Systems), 128-slice Definition Flash scanner (Siemens Healthcare), 192-slice Somatom Force CT scanner (Siemens Healthcare), 64-slice scanner (Definition Flash, Siemens Healthcare), 64-slice Siemens Sensation 64 (Siemens Healthcare), 128-slice scanner (Somatom Definition Flash, Siemens Healthcare), 64 detector row scanner (Brilliance 64, Philips Medical Systems) or 320 detector row scanner (Aquilion ONE, Toshiba Medical Systems)<br>**Image acquisition method:** DNM<br>**Tube voltage:** DNM<br>**Tube current:** DNM<br>**Matrix size:** DNM<br>**Field of view:** DNM<br>**Slice thickness:** 0.625 mm<br>**Slice interval:** DNM	**Adherence to radiomics guidelines:** nil<br>**Feature extraction software:** PyRadiomics integrated into 3D Slicer<br>**Hardware**: DNM	**Segmentation**: manual segmentation of the PVAT. PVAT defined as voxels ranging from -190 to -30 HU located within a radial distance from the outer vessel wall equal to the diameter of the respective vessel<br>**Processing**: voxels discretised into 16 bins of 10 HU width. Wavelet transformation applied<br>**Features extracted:** shape, first order, GLCM, GLDM, GLRLM, GLSZM, NGTDM and higher order<br>**Feature selection:** features with ICC ≥0.9 retained. Spearman's rank correlation coefficient used to remove correlated features ($\varrho \geq 0.8$). Hierarchical clustering performed using the squared Euclidean distance and the Ward method<br>**Machine learning techniques:** random forest	**Performance assessment:** predictive value of the model assessed using AUC from ROC<br>**Internal validation:** dataset split into training set and validation set in a ratio of 4:1. Fivefold cross validation performed<br>**External validation performed on the validation dataset**
**Study 3**	**CT coronary angiography** using 50-70 ml of iopamidol injected at 5.5-6.5 ml/s			

	**Scanner:** 64-slice scanner (General Electric, LightSpeed Ultra, General Electric)<br>**Image acquisition method:** DNM<br>**Tube voltage:** DNM<br>**Tube current:** DNM<br>**Matrix size:** DNM<br>**Field of view:** DNM<br>**Slice thickness:** 0.625 mm<br>**Slice interval:** DNM			
**Si et al.**[32]	**CT coronary angiography** using 60-80 ml of iodinated contrast injected at 4.5 ml/s<br>**Scanner:** 320-detector-row CT scanner (Aquilion ONE ViSION, Canon Medical Systems)<br>**Image acquisition method:** DNM<br>**Tube voltage:** 80-120 kV<br>**Tube current:** automatic modulation<br>**Matrix size:** DNM<br>**Field of view:** DNM<br>**Slice thickness:** 0.5 mm<br>**Slice interval:** 0.5 mm	**Adherence to radiomics guidelines:** nil<br>**Feature extraction software:** Research Portal (version 1.1, United Imaging Intelligence, Co., Ltd.)<br>**Hardware**: DNM	**Segmentation**: automated segmentation using the VB-net model. Tracking technology was used to connect the broken blood vessels that were segmented to improve the integrity of the blood vessel segmentation. Graph convolutional network-based point cloud for coronary artery segmentation and labelling was used to construct the key topological information of the coronary vascular tree. No assessment of the accuracy of the automated segmentation software<br>**Processing**: voxels resampled to 1.0 mm × 1.0 mm × 1.0 mm. Image normalisation applied<br>**Features extracted:** shape, first order, GLCM, GLRLM, GLSZM, GLDM and NGTDM<br>**Feature selection:** mRMR used to select 30 features. Then LASSO regression applied to select the most significant features<br>**Machine learning techniques:** logistic regression	**Performance assessment:** AUC from the ROC<br>**Internal validation:** dataset split into training set and validation set in a ratio of 7:3. Fivefold cross validation performed<br>**No external validation**
**Wen et al.**[33]	**CT coronary angiography**<br>**Scanner:** second- generation 128-section dual-source CT system (Somatom Definition Flash, Siemens Healthineers**)**<br>**Image acquisition method:** DNM	**Adherence to radiomics guidelines:** nil<br>**Feature extraction software:** PyRadiomics	**Segmentation**: manual segmentation of the PCAT using 3D slicer. PCAT was defined as all voxels in the range of -190 to -30 HU<br>**Processing**: images discretised to bin width 25 HU<br>**Features extracted:** first order, GLCM, GLRLM, GLSZM, GLDM and higher order	**Performance assessment:** AUC from the ROC<br>**Internal validation:** dataset split into training set and

	**Tube voltage:** 100 kV<br>**Tube current:** 370 mA<br>**Matrix size:** DNM<br>**Field of view:** DNM<br>**Slice thickness:** DNM<br>**Slice interval:** DNM	integrated into 3D Slicer (version 4.10.2)<br>**Hardware**: DNM	**Feature selection:** features with ICC > 0.75 retained. Univariate logistic analysis used to identify features that were able to significantly (P < 0.05) distinguish significant coronary artery stenosis from non-significant stenosis. Then, the Boruta algorithm was applied to select the most important features<br>**Machine learning techniques:** logistic regression, decision tree and SVM	validation set in a ratio of 4:1<br>**No external validation**
**You et al.**[34]	**CT coronary angiography** using iopromide injected at 4.5-5.5 ml/s<br>**Scanner:** 2 56-slice CT scanner (Brilliance iCT, Philips Medical Systems)<br>**Image acquisition**<br>**Tube voltage:** 100-120 kV<br>**Tube current:** automatically adjusted<br>**Matrix size:** DNM<br>**Field of view:** DNM<br>**Slice thickness:** 0.9 mm<br>**Slice interval:** 0.45 mm	**Adherence to radiomics guidelines:** nil<br>**Feature extraction software:** Artificial Intelligence Kit (GE Healthcare)<br>**Hardware**: DNM	**Segmentation**: semi-automated segmentation of the epicardial adipose tissue. Manual segmentation of the pericardial adipose tissue. The automated segmentation undertaken on EATseg software, and then manually adjusted on 3D slicer (version 4.11) by two radiologists. Epicardial adipose tissue was defined as voxels in the range of -190 to -30 HU. No assessment of the accuracy of the automated segmentation software<br>**Processing:** nil<br>**Features extracted:** first order, GLCM, GLSZM, GLRLM, NGTDM and GLDM<br>**Feature selection:** mRMR used to select 30 features. Then gradient boosting decision tree applied to filter features according to importance and those with a significant value for predicting MACE were retained<br>**Machine learning techniques:** logistic regression	**Performance assessment:** AUC from the ROC<br>**Internal validation:** dataset split into training set and validation set in a ratio of 7:3<br>**No external validation**
**Yu et al.**[35]	**CT coronary angiography** using 20 ml of iopromide injected at 4-5 ml/s<br>**Scanner:** 128-slice multidetector CT (Definition AS+, Siemens Healthineers)<br>**Image acquired** 4 seconds after the contrast bolus reached the ascending aorta<br>**Tube voltage:** 120 kV	**Adherence to radiomics guidelines:** nil<br>**Feature extraction software:** PyRadiomics integrated into an in-house software<br>**Hardware**: DNM	**Segmentation**: automated segmentation using CoronaryDoc, FAI Analysis Tool (version 5.1.2, ShuKun Technology). The software employs the skeleton erosion shrinkage algorithm to establish the centre line of each coronary branch. Along the centreline of the three major vessels, the normal sections of the blood vessels were cut at equal intervals and stacked in sequence to reconstruct a three-dimensional straightened blood vessel image. Then a sliding window was used to select	**Performance assessment:** AUC from the ROC<br>**Internal validation:** dataset split into training set and validation set in a ratio of 2:1. Fivefold cross validation

	**Tube current:** DNM<br>**Matrix size:** DNM<br>**Field of view:** DNM<br>**Slice thickness:** DNM<br>**Slice interval:** DNM	the starting and ending positions of the PCAT along the vessel. PCAT was defined as voxels in the range of -190 to -30 HU. No assessment of the accuracy of the automated segmentation software<br>**Processing**: DNM<br>**Features extracted:** first order, GLCM, GLSZM, GLRLM, NGTDM and GLDM<br>**Feature selection:** features with ICC > 0.85 retained. Then Spearman's rank correlation applied to assess correlation between the radiomics features and fractional flow reserve-based stenosis label. Features with low correlation or P value > 0.05 were removed. Next, LASSO regression applied to select the best features<br>**Machine learning techniques:** SVM	performed applied to training set<br>**No external validation**

Abbreviations: CT = computed tomography, HU = Hounsfield units, kV = kilovolts, mA = milliamperes, mm = millimetres, PVAT = peri-vascular adipose tissue, 3D = 3 dimensional, CTA = computed tomography angiogram, GLCM = gray level co-occurrence matrix, GLDM = gray level dependence matrix, GLSZM = gray level size zone matrix, GLRLM = gray level run length matrix, NGTDM = neighbouring gray tone difference matrix, ICC = intraclass correlation coefficient, LASSO = least absolute shrinkage and selection operator, SVM = support vector machine, AUC = area under curve, ROC = receiver operating characteristic, PPV = positive predictive value, NPV = negative predictive value, DNM = does not mention, IBSI = Image Biomarker Standardisation Initiative, CART = classification and regression tree, ANOVA = analysis of variance, MRMR minimum redundancy maximum relevance, PET = positron emission tomography, mCi = millicurie, kg = kilogram, ^{18}F-FDG = [^{18}F]Fluorodeoxyglucose, ROI = region of interest, SUV = standard uptake value, PCAT = peri-coronary adipose tissue, Light GBM = Light Gradient Boosting Machine, IVOCT = intra-vascular ocular coherence tomography, MBq = megabecquerel,^{18}F-NaF = [^{18}F]Sodiumfluoride, MACE = major adverse cardiovascular events.

Supplementary Table S4: The radiomics analysis and comparative analysis in the studies.

Study	Radiomics model	Comparator model(s)
**Carotid studies**		
**Chen et al.**[14]	Various models using carotid plaque radiomic features, PVAT radiomic features, and a combination of both to distinguish between symptomatic and asymptomatic plaques<br>Symptomatic plaques were defined as causing acute ischemic stroke or transient ischemic attack in the cerebral anterior circulation region within 2 weeks prior to the CT angiogram	Model consisting of CT angiogram features (plaque ulceration (defined as extension of contrast > 1.5 mm beyond the vessel lumen), remodelling index (defined as area of the vessel at the maximum stenosis ÷ area of the distal vessel unaffected by plaque × 100%)) and a combined model incorporating CT angiography features with radiomic features
**Cilla et al.**[15]	Model to differentiate between vulnerable and non-vulnerable carotid plaques<br>Plaque vulnerability assessed using histological assessment: vulnerable plaque defined as containing atheromatous debris or demonstrating intraplaque haemorrhage; non-vulnerable plaques defined as containing calcified or with collagen-rich sclerotic tissue	Nil
**Ebrahimian et al.**[26]	Two models using spectral radiomic features or single-energy radiomic features to differentiate between different grades of ICA/CCA stenosis and prediction of surgical outcomes	Model consisting of spectral segmentation features: mean mixed CT values (mean HU from mixed volume DECT images), mean iodine CT values (mean HU from material density iodine images), mean Vnc CT values (mean mixed HU - mean iodine HU), total iodine uptake, total iodine concentration (iodine within per unit volume in mg/mL), vital iodine uptake (iodine uptake within the part of the ROI that excludes the non-enhancing portion), and vital iodine concentration (iodine concentration within the part of the ROI that excludes the non-enhancing portion).
**Kafouris et al.**[36]	Model based on ^{18}F-FDG PET/CT radiomic features to predict event-prone carotid plaques in patients with high-grade carotid stenosis.<br>Event-prone plaques were defined using histological (extent of lipid core and severity of calcification) and immunohistochemical analysis (CD31 antibody density and CD68 antibody density)	Model consisting of radiotracer uptake measurements: SUV and TBR

**Liu et al.**[37]	Model using carotid plaque radiomic features to distinguish between symptomatic and asymptomatic patients. Symptomatic defined as a stroke or TIA occurring in the carotid supplying territory	Model consisting of CT angiography features (presence of calcification, maximum plaque thickness, classification of luminal stenosis using NASCET criteria) and a combined model incorporating CT angiography features with radiomic features
**Nie et al.**[38]	Model using PVAT radiomic features to differentiate between symptomatic and asymptomatic carotid plaques. Symptomatic defined as causing TIA, anterior circulation stroke, or monocular symptoms 2 weeks prior to the CT angiography and/or an MRI head demonstrating acute/subacute stroke	Model consisting of CT angiography features (plaque thickness, plaque length, remodelling index (the average of the maximum external vessel diameter of the plaque over the normal diameter of the proximal and distal regions), plaque ulceration (spread of contrast deep into the plaque), high risk plaque (having ≥ 2 of positive remodelling index > 1.1, punctate calcification, or low-density plaque), and napkin ring sign) and a combined model incorporating CT angiography features with radiomic features
**Le et al.**[39]	Model using plaque radiomic features to differentiate between culprit and non-culprit carotid artery lesions. Culprit artery defined as the carotid laterality consistent with the stroke or TIA symptoms	Model consisting of CT angiography features: Agatston score
**Shan et al.**[40]	Model using plaque radiomic features to identify vulnerable carotid plaques. Vulnerable plaques defined as observing contrast agent in the plaque indicating neo-vascularisation	Nil
**Shi et al.**[41]	Model using plaque radiomic features to identify symptomatic carotid plaques. Symptomatic defined as acute/subacute stroke findings on MRI in the ipsilateral carotid artery territory within 2 weeks before imaging scans	Model consisting of CT angiography and clinical features (plaque ulceration (defined as ≥ 1 mm contrast entering the plaque in a single plane), carotid rim sign defined as adventitial calcification (< 2 mm thickness) with interior soft plaque (>2mm thickness)) and homocysteine levels) and a combined model incorporating CT angiography and clinical features with radiomic features
**Xia et al.**[42]	Model using plaque radiomic features to predict the risk of TIA events in patients with carotid artery stenosis of 30–50%	Model consisting of clinical features such as blood tests (triglycerides, low-density lipoprotein, homocysteine, uric acid, and fibrinogen), demographics (age, gender and BMI), blood pressure, diabetes, smoking, stenosis of the carotid artery, and current medications (antiplatelets and lipid-lowering drugs), and a combined model incorporating clinical features with radiomic features
**Coronary studies**		

**Chen et al.**[16]	Model using PCAT radiomic features to predict obstructive coronary artery disease in patients with T2DM	Model consisting of CTCA features: Agatston score quantified, stenosis classified using the SCCT coronary 18-segmentation classification criteria[31]
**Chen et al.**[17]	Model using plaque radiomic features to predict rapid plaque progression in coronary arteries. Rapid plaque progression defined as an annual increase > 1% on follow-up CTCA scans	Model consisting of CTCA features (calcific volume, fibrotic volume and low attention plaque volume) and a combined model incorporating CTCA features with radiomic features
**Feng et al.**[18]	Model using plaque radiomic features to predict plaque progression in coronary arteries. Plaque progression defined as the annual change in plaque burden	Model consisting of CTCA features (non-calcified plaque burden and fat attenuation index) and a combined model incorporating CTCA features with radiomic features
**Homayounieh et al.**[19]	Model using plaque radiomic features from single-click whole heart non-contrast CT images to predict coronary artery stenosis and 10-year MESA CHD risk	Models consisting of clinical features (demographics, BMI, blood pressure, smoking history, co-morbidities (T2DM, hypertension, hyperlipidemia, heart disease and cerebrovascular accidents), family history of heart disease, blood glucose and lipid profile) and combined models incorporating clinical features with radiomic features
**Hou et al.**[20]	Model using PCAT radiomic features to predict myocardial ischaemia	Various models incorporating different combinations of CTCA features (lesion length, maximum stenosis diameter, maximum stenosis area), CT-fractional flow reserve and pericoronary fat attenuation index
**Hu et al.**[21]	Model using coronary plaque radiomic features to predict myocardial ischaemia and MACEs	Model consisting of CTCA features: Agatston score, lesion length, diameter stenosis and high-risk plaque characteristics (defined as ≥2 of low attenuation plaques, positive remodelling, spotty calcification, and napkin-ring sign)
**Jing et al.**[22]	Model using PCAT radiomic feature to predict ACS in patients with ACS, chronic CAD and no CAD	Model consisting of CTCA features: FAI value of the right coronary artery
**Kim et al.**[23]	Model using PCAT radiomic features to identify plaques with microscopic features of vulnerability (thin-cap fibroatheroma and microchannels) that have been confirmed using intravascular optical coherence tomography	Nil
**Kwiecinski et al.**[24]	Model using plaque radiomic features from ^{18}F-NaF PET/CT and CTCA to predict MI	Nil
**Lee et al.**[25]	Model of plaque radiomic features in normal coronary arteries to predict the development of coronary plaques	Model consisting of CTCA features (plaque length and total plaque volume) and clinical features (age, gender, BMI, systolic blood pressure, smoking history, hypertension, T2DM, family history of heart disease,

		low-density lipoprotein cholesterol level, medication use) and a combined model incorporating CTCA and clinical features with radiomic features
**Li et al.**[27]	Model using plaque radiomic features in patients with CAD and end-stage CHF to identify vulnerable plaques. Vulnerable plaque defined as having ≥ 2 of active inflammation, fibrous cap thickness < 65 μm and lipid core of > 40% of plaque total area, endothelial denudation with superficial platelet aggregation or plaque erosion, fissured or injured plaque, or intraplaque haemorrhage on histological assessment	Model consisting of CTCA features: low attenuation plaque (3 regions of interest < 30 HU), positive remodelling (remodelling index > 1.1), spotty calcification (presence of calcified plaque with diameter <3 mm), napkin-ring sign
**Li et al.**[28]	Model using plaque radiomic features to distinguish between chronic total occlusion and subtotal occlusion. The degree of occlusion was assessed using invasive angiography	Model consisting of CTCA features (total lesion length, transluminal attenuation gradient - defined as the change in HU per 10 mm of coronary artery length, remodelling - defined as ratio of >1 of the diameter of the occluded vessel to the adjacent normal vessel, classification of plaque calcification (low attenuation = -30-30 HU, non-calcified = 31-350 HU, calcified = > 350 HU)) and a combined model incorporating CTCA features with radiomic features
**Lin et al.**[29]	Model using PCAT radiomic features to distinguish between acute MI, stable CAD and no CAD	Various models incorporating different combinations of clinical features (age, gender, T2DM, hypertension, smoking status, serum lipid levels, and CRP), CTCA features (PCAT attenuation values) and radiomic features
**Lin et al.**[30]	Model combining plaque radiomic features and CTCA features to distinguish between culprit and non-culprit lesions in patients with acute MI and in patients with stable CAD. Culprit lesions confirmed using invasive coronary angiography	Model consisting of CTCA features only: positive remodelling, low attenuation plaque, spotty calcification, napkin-ring sign, total plaque volume, volumes and compositions of non-calcified plaque and low-density non-calcified plaque
**Oikonomou et al.**[31]	Study 2: model using PVAT radiomic features to distinguish between MACE and non-MACE cases<br>Study 3: model using PVAT radiomic features to identify changes in the adipose tissue related to acute MI over 6 months when compared to stable CAD patients	Study 2: nil<br>Study 3: model using perivascular fat attenuation index from CTCA
**Si et al.**[32]	Model using PCAT radiomic features to distinguish between acute MI and unstable angina cases	Models consisting of CTCA features (fat attenuation index) and a combined model incorporating CTCA features with radiomic features

**Wen et al.**[33]	Model using PCAT radiomic features to identify haemodynamically significant coronary artery stenosis. Coronary artery stenosis confirmed using fractional flow reserve from invasive coronary angiography	Models consisting of CTCA features (Agatston score and diameter stenosis) and a combined model incorporating CTCA features with radiomic features
**You et al.**[34]	Model using PCAT and EAT radiomic features to predict MACE within 3 years	Various models consisting of combinations of clinical features (serum cholesterol, serum low-density lipoprotein cholesterol, and serum triglycerides) only, clinical features with PCAT radiomic features and clinical features with EAT radiomic features
**Yu et al.**[35]	Model using PCAT radiomic features to identify significant coronary artery stenosis. Coronary artery stenosis confirmed using invasive coronary angiography	Model consisting of CT-FFR values

Abbreviations: PVAT = peri-vascular adipose tissue, CT = computed tomography, ICA = internal carotid artery, CCA = common carotid artery, HU = Hounsfield units, DECT = dual-energy computed tomography, ROI = region of interest,, FDG = Fluorodeoxyglucose, PET = positron emission tomography, SUV = standard uptake value, TBR = target to background ratio, TIA = transient ischaemic attack, NASCET = North American Symptomatic Carotid Endarterectomy Trial, MRI = magnetic resonance imaging, BMI = body mass index, PCAT = pericoronary adipose tissue, T2DM = type 2 diabetes mellitus, CTCA = computed tomography coronary angiogram, SCCT = Society of Cardiovascular Computed Tomography, MESA = multi-ethnic study of atherosclerosis, CHD, = coronary heart disease, MACE = major adverse cardiovascular event, ACS = acute coronary syndrome, CAD = coronary artery disease, FAI = fat attenuation index, MI = myocardial infarction, CHF = congestive heart failure, CRP = C reactive protein, EAT = epicardial adipose tissue, FFR = fractional flow reserve.
